# Supplementary material for: Lower free triiodothyronine (fT3) levels in cirrhosis are linked to systemic inflammation, higher risk of acute-on-chronic liver failure, and mortality
Source: JHEP Rep. 2023 Nov 1;6(1):100954. doi: 10.1016/j.jhepr.2023.100954 (PMC10733101; doi:10.1016/j.jhepr.2023.100954)
Supplement: Multimedia component 1 [file mmc1.pdf]

Lower free triiodothyronine (fT3) levels in cirrhosis are linked to systemic inflammation, higher risk of acute-on-chronic liver failure, and mortality

Lukas Hartl, Benedikt Simbrunner, Mathias Jachs, Peter Wolf, David Josef Maria Bauer, Bernhard Scheiner, Lorenz Balcar, Georg Semmler, Michael Schwarz, Rodrig Marculescu, Varius Dannenberg, Michael Trauner, Mattias Mandorfer, Thomas Reiberger

Table of contents

Table S1………………………………………………………………………………2
Table S2………………………………………………………………………………3
Table S3………………………………………………………………………………4
Table S4………………………………………………………………………………5
Table S5………………………………………………………………………………6
Table S6………………………………………………………………………………7
Table S7………………………………………………………………………………9
Table S8……………………………………………………………………………..…11
Table S9……………………………………………………………………………..…13
Table S10…………………………………………………………………………..…..14
Table S11…………………………………………………………………………..…..15

Table-S1. Levels of pituitary-thyroid axis parameters, parameters of liver function and systemic inflammation in patients with different stages of advanced chronic liver disease

Parameter, Unit	EASL stage							p-value
	pACLD (n=10)	0 (n=33)	1 (n=42)	2 (n=44)	3 (n=12)	4 (n=97)	5 (n=59)	
**TSH, µIU x mL[1] (IQR)**	1.2 (0.7-2.3)	1.2 (1.0-1.7)	1.5 (0.8-2.1)	1.4 (1.1-2.1)	1.6 (0.8-2.7)	1.7 (1.2-2.4)	1.5 (1.0-2.3)	0.152
**Elevated TSH, n (%)**	0 (0.0%)	1 (3.0%)	2 (4.8%)	0 (0.0%)	0 (0.0%)	3 (3.1%)	3 (3.1%)	0.765
**fT4, ng x dL^{-1} (IQR)**	1.2 (1.0-1.3)	1.1 (1.1-1.3)	1.1 (1.0-1.2)	1.1 (1.0-1.3)	1.2 (1.0-1.3)	1.2 (1.0-1.3)	1.2 (1.1-1.3)	0.338
**Low fT4, n (%)**	0 (0.0%)	1 (3.0%)	0 (0.0%)	0 (0.0%)	0 (0.0%)	1 (1.0%)	1 (1.7%)	0.842
**fT3, pg x mL^{-1} (IQR)**	3.2 (3.1-3.5)	3.0 (2.7-3.3)	2.9 (2.5-3.2)	2.9 (2.6-3.2)	2.9 (2.3-3.3)	2.7 (2.3-3.0)	2.5 (2.1-2.8)	**<0.001**
**Low fT3, n (%)**	0 (0.0%)	2 (6.1%)	4 (9.5%)	3 (6.8%)	1 (8.3%)	12 (12.4%)	16 (27.1%)	**0.015**
**CTP score, points (IQR)**	5 (5-6)	5 (5-5)	5 (5-6)	5 (5-6)	5 (5-6)	7 (6-9)	8 (7-10)	**<0.001**
**MELD, points (IQR)**	8 (7-12)	8 (7-11)	9 (8-14)	11 (9-14)	11 (10-15)	14 (10-17)	14 (10-18)	**<0.001**
**Bilirubin, mg x dL^{-1} (IQR)**	0.7 (0.5-1.4)	0.8 (0.5-1.0)	0.8 (0.6-1.4)	1.3 (0.8-2.3)	1.0 (0.8-1.9)	1.3 (0.8-2.7)	1.7 (0.9-2.5)	**<0.001**
**Albumin, g x dL^{-1} (IQR)**	39.9 (37.0-41.5)	41.0 (38.9-43.0)	38.4 (34.3-42.4)	37.8 (35.6-40.4)	39.5 (37.7-40.7)	35.4 (31.1-38.5)	34.1 (29.9-37.7)	**<0.001**
**IL-6, ng x dL^{-1} (IQR) [1]**	4.6 (3.5-6.2)	4.2 (2.7-7.1)	5.8 (2.6-13.1)	6.1 (4.1-9.6)	6.6 (5.0-13.4)	10.5 (6.7-18.5)	13.5 (8.7-24.4)	**<0.001**
**CRP, mg x dL^{-1} (IQR)**	0.2 (0.0-0.2)	0.1 (0.1-0.3)	0.2 (0.1-0.7)	0.2 (0.1-0.3)	0.1 (0.1-0.3)	0.4 (0.2-0.8)	0.4 (0.2-0.7)	**<0.001**

[1] available in 286 patients (stage pACLD: n=10; 0: n=33; stage 1: n=41; stage 2: n=40; stage 3: n=12; stage 4: n=94; stage 5: n=56)

Table-S2. Levels of thyroid stimulating hormone (TSH) and free triiodothyronine (fT3) in different strata of body mass index (BMI) among patients with compensated (cACLD) and decompensated advanced chronic liver disease (dACLD)

	cACLD (n=129)				dACLD (n=168)			
**BMI strata**	**<25 kg/m^2**	**25-30 kg/m^2**	**>30 kg/m^2**	**p-value**	**<25 kg/m^2**	**25-30 kg/m^2**	**>30 kg/m^2**	**p-value**
**Number of patients, n (%)**	48 (37.2%)	39 (30.2%)	42 (32.6%)		81 (48.2%)	58 (34.5%)	29 (17.3%)	
**TSH, µIU/mL (IQR)**	1.3 (0.9-2.3)	1.3 (1.0-1.7)	1.4 (1.1-1.8)	0.844	1.7 (1.1-2.6)	1.4 (1.1-2.0)	2.0 (1.2-3.0)	0.514
**fT3, pg/mL (IQR)**	3.0 (2.6-3.3)	2.9 (2.6-3.1)	3.0 (2.7-3.3)	0.222	2.7 (2.2-3.0)	2.6 (2.4-3.0)	2.6 (2.2-2.9)	0.381

Table-S3. Correlations of pituitary-thyroid axis parameters, parameters of liver function, endothelial dysfunction, hyperdynamic circulation and systemic inflammation in patients advanced chronic liver disease

	fT3, pg x mL^{-1}		fT4, ng x dL^{-1}		TSH, µIU x mL^{-1}	
	rho	p-value	rho	p-value	rho	p-value
**TSH, µIU x mL^{-1}**	-0.152	0.009	-0.109	0.061	-	-
**fT4, ng x dL^{-1}**	0.000	0.998	-	-	-0.109	0.061
**fT3, pg x mL^{-1}**	-	-	0.000	0.998	-0.152	0.009
**MELD, points**	-0.448	<0.001	0.060	0.300	0.132	0.023
**Albumin, mg x L^{-1}**	0.434	<0.001	-0.012	0.835	-0.179	0.002
**HVPG, mmHg**	-0.300	<0.001	0.069	0.233	0.152	0.009
**VWF, %**	-0.357	<0.001	0.046	0.431	0.024	0.687
**proBNP, pg x mL^{-1}**	-0.385	<0.001	0.226	<0.001	0.107	0.070
**IL-6, ng x dL^{-1} (IQR) [3]**	-0.496	<0.001	0.146	0.013	0.222	<0.001
**CRP, mg x dL^{-1}**	-0.356	<0.001	0.188	0.001	0.113	0.052

Table-S4. Impact of parameters of hepatic dysfunction, kidney function and systemic inflammation on (i) free triiodothyronine (fT3) and (ii) thyroid stimulating hormone (TSH) levels. Univariate and multivariate multivariate linear regression models are shown.

Parameter, Unit	Univariate (unadjusted) analysis			Multivariate (adjusted) analysis		
**(i) fT3, pg/mL**	**B**	**95%CI**	**p-value**	**aB**	**95%CI**	**p-value**
**Age, 10 years**	-0.10	-0.16 - -0.03	**0.005**	-0.11	-0.17 – -0.05	**<0.001**
**Sex (male)**	0.10	-0.08 - 0.28	0.276	-	-	-
**Body Mass Index, kg x m^{-2}**	0.01	-0.01 - 0.02	0.621	-0.01	-0.02 - 0.01	0.495
**Child Turcotte Pugh score, points**	-0.16	-0.20 - -0.12	**<0.001**	-0.15	-0.19 - -0.11	**<0.001**
**Creatinine, mg x dL^{-1}**	-0.08	-0.17 - 0.01	0.074	-0.06	-0.15 – 0.02	0.129
**Sodium, mmol x L^{-1}**	0.04	0.02 - 0.06	**<0.001**	-0.01	-0.04 - 0.01	0.279
**HVPG, mmHg**	-0.03	-0.04 - -0.02	**<0.001**	-0.01	-0.02 - 0.00	0.161
**C-reactive protein, mg x dL^{-1}**	-0.42	-0.59 - -0.25	**<0.001**	-0.17	-0.34 - -0.01	**0.048**
**(ii) TSH, µIU/mL**	**B**	**95%CI**	**p-value**	**aB**	**95%CI**	**p-value**
**Age, 10 years**	-0.01	-0.17 - 0.17	0.955	-	-	-
**Sex (male)**	-0.21	-0.66 - 0.25	0.371	-	-	-
**Body Mass Index, kg x m^{-2}**	-0.01	-0.05 – 0.03	0.690	0.00	-0.04 – 0.04	0.935
**Child Turcotte Pugh score, points**	0.07	-0.04 - 0.18	0.190	0.02	-0.11 - 0.14	0.779
**Creatinine, mg x dL^{-1}**	0.07	-0.16 - 0.30	0.533	-	-	-
**Sodium, mmol x L^{-1}**	0.02	-0.03 - 0.08	0.426	-	-	-
**HVPG, mmHg**	0.03	-0.01 - 0.06	0.113	0.02	-0.02 - 0.05	0.318
**C-reactive protein, mg x dL^{-1}**	0.45	0.01 - 0.89	**0.046**	0.45	0.01 - 0.89	**0.046**

Table-S5. Impact of parameters of hepatic dysfunction, kidney function and systemic inflammation on (i) free triiodothyronine (fT3) and (ii) thyroid stimulating hormone (TSH) levels in patients with decompensated advanced chronic liver disease (dACLD).

Univariate and multivariate multivariate linear regression models are shown.

Parameter, Unit	Univariate (unadjusted) analysis			Multivariate (adjusted) analysis		
**(i) fT3, pg/mL**	**B**	**95%CI**	**p-value**	**aB**	**95%CI**	**p-value**
**Age, 10 years**	-0.04	-0.11 - 0.02	0.200	-	-	-
**Sex (male)**	0.13	-0.05 - 0.31	0.164	-	-	-
**Body Mass Index, kg x m^{-2}**	0.00	-0.02 - 0.02	0.950	-0.01	-0.02 - 0.01	0.465
**Child Turcotte Pugh score, points**	-0.13	-0.17 - -0.09	**<0.001**	-0.11	-0.15 - -0.07	**<0.001**
**Creatinine, mg x dL^{-1}**	-0.10	-0.18 - -0.01	**0.023**	-0.08	-0.15 – 0.00	**0.049**
**Sodium, mmol x L^{-1}**	0.02	0.00 - 0.04	0.058	-0.01	-0.03 - 0.01	0.178
**HVPG, mmHg**	-0.02	-0.04 - -0.01	**0.008**	-0.01	-0.02 - 0.00	0.167
**C-reactive protein, mg x dL^{-1}**	-0.32	-0.47 - -0.18	**<0.001**	-0.19	-0.33 - -0.05	**0.008**
**(ii) TSH, µIU/mL**	**B**	**95%CI**	**p-value**	**aB**	**95%CI**	**p-value**
**Age, 10 years**	0.00	-0.28 - 0.27	0.978	-	-	-
**Sex (male)**	-0.46	-1.21 - 0.28	0.223	-	-	-
**Body Mass Index, kg x m^{-2}**	-0.01	-0.08 – 0.06	0.839	0.00	-0.07 – 0.07	0.912
**Child Turcotte Pugh score, points**	0.03	-0.14 - 0.21	0.719	0.02	-0.16 - 0.20	0.799
**Creatinine, mg x dL^{-1}**	0.10	-0.25 - 0.45	0.571	-	-	-
**Sodium, mmol x L^{-1}**	0.07	-0.02 - 0.14	0.129	-	-	-
**HVPG, mmHg**	0.02	-0.05 - 0.08	0.562	0.02	-0.05 - 0.08	0.562
**C-reactive protein, mg x dL^{-1}**	0.43	-0.21 - 1.06	0.185	-	-	-

Table-S6. Impact of thyroid stimulating hormone (TSH) on the risk of (i) decompensation/further decompensation, (ii) acute-on-chronic liver failure (ACLF) and (iii) liver-related death. Univariate and multivariate multivariate competing risk regression models are shown. Etiological cure, hepatocellular carcinoma, liver transplantation and non-liver-related death were considered as competing risks.

**Parameter, Unit**	**Univariate (unadjusted) analysis**			**Multivariate (adjusted) analysis**		
**(i) Outcome: (further) decompensation**	**sHR**	**95%CI**	**p-value**	**asHR**	**95%CI**	**p-value**
**TSH, µIU x mL^{1}**	1.02	0.94-1.10	0.660	1.00	0.90-1.10	0.930
**Age, 10 years**	0.98	0.81-1.19	0.840	-	-	-
**Sex (male)**	0.90	0.54-1.51	0.690	-	-	-
**Body Mass Index, kg x m^{-2}**	0.99	0.93-1.05	0.720	1.03	0.96-1.10	0.470
**Child Turcotte Pugh score, points**	1.26	1.13-1.39	**<0.001**	1.19	1.03-1.37	**0.019**
**Creatinine, mg x dL^{-1}**	0.83	0.60-1.16	0.280	-	-	-
**Sodium, mmol x L^{-1}**	0.93	0.89-0.98	**0.009**	1.01	0.94-1.08	0.810
**HVPG, mmHg**	1.10	1.07-1.14	**<0.001**	1.09	1.04-1.14	**<0.001**
**C-reactive protein, mg x dL^{-1}**	1.49	1.01-2.19	**0.042**	0.85	0.52-1.39	0.530
**(ii) Outcome: ACLF**	**sHR**	**95%CI**	**p-value**	**asHR**	**95%CI**	**p-value**
**TSH, µIU x mL^{1}**	1.05	0.94-1.17	0.400	1.07	0.94-1.22	0.290
**Age, 10 years**	1.27	0.83-1.94	0.270	-	-	-
**Sex (male)**	1.02	0.39-2.65	0.970	-	-	-
**Body Mass Index, kg x m^{-2}**	0.95	0.78-1.15	0.610	1.01	0.81-1.26	0.940
**Child Turcotte Pugh score, points**	1.54	1.28-1.86	**<0.001**	1.36	1.07-1.74	**0.013**
**Creatinine, mg x dL^{-1}**	1.23	1.06-1.43	**0.007**	1.00	0.84-1.20	0.970
**Sodium, mmol x L^{-1}**	0.85	0.79-0.92	**<0.001**	0.93	0.85-1.03	0.170
**HVPG, mmHg**	1.07	0.92-1.25	0.390	1.09	1.00-1.20	0.063
**C-reactive protein, mg x dL^{-1}**	2.21	1.21-4.02	**0.009**	0.99	0.51-1.94	0.980

(iii) Outcome: Liver-related death	sHR	95%CI	p-value	asHR	95%CI	p-value
**TSH, µIU x mL^{1}**	1.06	0.96-1.16	0.250	1.08	0.95-1.24	0.250
**Age, 10 years**	1.22	0.75-2.01	0.420	-	-	-
**Sex (male)**	0.78	0.27-2.30	0.650	-	-	-
**Body Mass Index, kg x m^{-2}**	0.91	0.79-1.05	0.210	0.98	0.81-1.18	0.810
**Child Turcotte Pugh score, points**	1.63	1.26-2.09	**<0.001**	1.42	0.99-2.04	0.054
**Creatinine, mg x dL^{-1}**	1.03	0.86-1.22	0.780	-	-	-
**Sodium, mmol x L^{-1}**	0.83	0.75-0.91	**<0.001**	0.90	0.79-1.04	0.160
**HVPG, mmHg**	1.15	1.07-1.24	**<0.001**	1.11	0.99-1.23	0.073
**C-reactive protein, mg x dL^{-1}**	2.21	1.12-4.35	**0.022**	0.84	0.38-1.84	0.660

Table-S7. Impact of free thyroxin (fT4) on the risk of (i) decompensation/further decompensation, (ii) acute-on-chronic liver failure (ACLF) and (iii) liver-related death. Univariate and multivariate multivariate competing risk regression models are shown. Etiological cure, hepatocellular carcinoma, liver transplantation and non-liver-related death were considered as competing risks.

**Parameter of interest**	**Univariate (unadjusted) analysis**			**Multivariate (adjusted) analysis**		
**(i) Outcome: (further) decompensation**	**sHR**	**95%CI**	**p-value**	**asHR**	**95%CI**	**p-value**
**fT4, ng x dL^{-1}**	1.12	0.52-2.42	0.780	0.92	0.38-2.23	0.860
**Age, 10 years**	0.98	0.81-1.19	0.840	-	-	-
**Sex (male)**	0.90	0.54-1.51	0.690	-	-	-
**Body Mass Index, kg x m^{-2}**	0.99	0.93-1.05	0.720	1.03	0.95-1.10	0.480
**Child Turcotte Pugh score, points**	1.26	1.13-1.39	**<0.001**	1.18	1.02-1.37	**0.024**
**Creatinine, mg x dL^{-1}**	0.83	0.60-1.16	0.280	-	-	-
**Sodium, mmol x L^{-1}**	0.93	0.89-0.98	**0.009**	1.01	0.94-1.08	0.850
**HVPG, mmHg**	1.10	1.07-1.14	**<0.001**	1.09	1.04-1.14	**<0.001**
**C-reactive protein, mg x dL^{-1}**	1.49	1.01-2.19	**0.042**	0.86	0.51-1.46	0.580
**(ii) Outcome: ACLF**	**sHR**	**95%CI**	**p-value**	**asHR**	**95%CI**	**p-value**
**fT4, ng x dL^{-1}**	1.33	0.31-5.64	0.700	0.42	0.08-2.16	0.300
**Age, 10 years**	1.27	0.83-1.94	0.270	-	-	-
**Sex (male)**	1.02	0.39-2.65	0.970	-	-	-
**Body Mass Index, kg x m^{-2}**	0.95	0.78-1.15	0.610	1.00	0.79-1.26	0.990
**Child Turcotte Pugh score, points**	1.54	1.28-1.86	**<0.001**	1.34	1.03-1.73	**0.027**
**Creatinine, mg x dL^{-1}**	1.23	1.06-1.43	**0.007**	0.97	0.80-1.17	0.710
**Sodium, mmol x L^{-1}**	0.85	0.79-0.92	**<0.001**	0.92	0.83-1.02	0.120
**HVPG, mmHg**	1.07	0.92-1.25	0.390	1.10	1.00-1.20	0.051

**C-reactive protein, mg x dL^{-1}**	2.21	1.21-4.02	**0.009**	1.17	0.56-2.45	0.680
**(iii) Outcome: Liver-related death**	**sHR**	**95%CI**	**p-value**	**asHR**	**95%CI**	**p-value**
**fT4, ng x dL^{-1}**	1.61	0.32-8.21	0.560	0.560	0.06-5.14	0.610
**Age, 10 years**	1.22	0.75-2.01	0.420	-	-	-
**Sex (male)**	0.78	0.27-2.30	0.650	-	-	-
**Body Mass Index, kg x m^{-2}**	0.91	0.79-1.05	0.210	0.97	0.82-1.15	0.710
**Child Turcotte Pugh score, points**	1.63	1.26-2.09	**<0.001**	1.40	0.93-2.10	0.110
**Creatinine, mg x dL^{-1}**	1.03	0.86-1.22	0.780	-	-	-
**Sodium, mmol x L^{-1}**	0.83	0.75-0.91	**<0.001**	0.90	0.76-1.06	0.200
**HVPG, mmHg**	1.15	1.07-1.24	**<0.001**	1.11	0.99-1.24	0.070
**C-reactive protein, mg x dL^{-1}**	2.21	1.12-4.35	**0.022**	0.96	0.35-2.64	0.940

Table-S8. Patient characteristics according to low vs. non-low free triiodothyronine (fT3) levels

Patient characteristics	Patients with low fT3 (n=38)	Patients without low fT3 (n=259)	p-value
**Sex, male/female (% male)**	18/20 (47.4%)	181/78 (69.9%)	**0.006**
**Age, years (IQR)**	55.3 (47.1-63.9)	56.0 (48.1-63.8)	0.915
**BMI, kg x m^{-2} (IQR)**	23.3 (20.7-26.3)	26.4 (23.5-30.2)	**0.003**
**Etiology**			0.156
**ALD, n (%)**	24 (63.2%)	136 (52.5%)	
**Viral hepatitis, n (%)**	2 (5.3%)	47 (18.1%)	
**MASH, n (%)**	2 (5.3%)	33 (12.7%)	
**Cholestatic, n (%)**	3 (7.9%)	18 (7.0)	
**AIH, n (%)**	2 (5.3%)	7 (2.7%)	
**Other, n (%)**	5 (13.2%)	18 (7.0%)	
**Decompensated ACLD, n (%)**	29 (76.3%)	139 (53.7%)	**0.009**
**Ascites, n (%)**	28 (73.7%)	116 (44.8%)	**<0.001**
**Refractory ascites, n (%)**	11 (28.9%)	16 (6.2%)	**<0.001**
**Hepatic encephalopathy, n (%)**	13 (34.2%)	41 (15.8%)	**0.006**
**Variceal bleeding, n (%)**	5 (13.2%)	30 (11.6%)	0.779
**EASL stage**			**0.015**
**probable ACLD, n (%)**	0 (0.0%)	10 (3.9%)	
**0, n (%)**	2 (5.3%)	31 (12.0%)	
**1, n (%)**	4 (10.5%)	38 (14.7%)	
**2, n (%)**	3 (7.9%)	41 (15.8%)	
**3, n (%)**	1 (2.6%)	11 (4.2%)	
**4, n (%)**	12 (31.6%)	85 (32.8%)	
**5, n (%)**	16 (42.1%)	43 (16.6%)	
**MELD, points (IQR)**	17 (14-19)	11 (9-14)	**<0.001**
**CTP score, points (IQR)**	9 (8-10)	6.0 (5-8)	**<0.001**

**CTP stage**			**<0.001**
**A, n (%)**	4 (10.6%)	155 (59.8%)	
**B, n (%)**	20 (52.6%)	82 (31.7%)	
**C, n (%)**	14 (36.8%)	22 (8.5%)	
**Bilirubin, mg x dL^{-1} (IQR)**	2.6 (1.7-4.3)	1.0 (0.7-2.0)	**<0.001**
**Albumin, g x dL^{-1} (IQR)**	31.9 (27.4-35.9)	37.4 (33.6-40.4)	**<0.001**
**INR, units (IQR)**	1.6 (1.3-1.8)	1.4 (1.2-1.5)	**0.039**
**Sodium, mmol x L^{-1} (IQR)**	136.0 (134.0-138.0)	139.0 (137.0-141.0)	**0.001**
**HVPG, mmHg (IQR)**	19 (15-24)	16 (11-20)	0.315
**MAP, mmHg (IQR)**	91 (82-106)	101 (91-111)	0.059
**LSM, kPa (IQR)**	57.3 (31.4-75.0)	35.6 (21.6-59.5)	**0.023**
**TSH, µIU x mL-1 (IQR)**	2.0 (1.3-2.6)	1.4 (1.0-2.2)	**0.023**
**fT3, pg x mL-1 (IQR)**	1.9 (1.7-2.0)	2.9 (2.6-3.2)	**<0.001**
**fT4, ng x dL-1 (IQR)**	1.2 (1.1-1.3)	1.2 (1.0-1.3)	0.612
**IL-6, ng x dL^{-1} (IQR)**	22.0 (13.3-27.4)	7.3 (4.3-13.1)	**<0.001**
**WBC, G x L^{-1} (IQR)**	4.6 (3.3-6.5)	4.8 (3.5-6.3)	0.999
**CRP, mg x dL^{-1} (IQR)**	0.5 (0.2-1.5)	0.2 (0.1-0.6)	**<0.001**

Table-S9. Clinical outcomes of patients according to low vs. non-low free triiodothyronine (fT3)

Clinical outcomes	Patients with low fT3 (n=38)	Patients with non-low fT3 (n=259)	p-value
**Follow-up time, days (IQR)**	292.0 (154.0-604.0)	427.0 (156.0-823.0)	0.233
**Decompensation event, n (%)**	14 (36.8%)	61 (23.6%)	0.078
**Ascitic complication, n (%)**	4 (10.5%)	39 (15.1%)	0.458
**Variceal bleeding, n (%)**	3 (7.9%)	10 (3.9%)	0.256
**Hepatic encephalopathy, n (%)**	2 (5.3%)	37 (14.3%)	0.124
**ACLF, n (%)**	11 (28.9%)	15 (5.8%)	**<0.001**
**Bacterial infections, n (%)[1]**	5 (13.5%)	33 (13.2%)	0.958
**SBP, n (%)**	3 (7.9%)	5 (1.9%)	**0.034**
**Acute kidney injury, n (%)[1]**	5 (13.5%)	19 (7.6%)	0.225
**HCC, n (%)**	0 (0.0%)	10 (3.9%)	0.218
**TIPS implantation, n (%)**	4 (10.5%)	13 (5.0%)	0.172
**Liver transplantation, n (%)**	4 (10.5%)	15 (5.8%)	0.265
**Death, n (%)**	11 (28.9%)	22 (8.5%)	**<0.001**
**Liver-related death, n (%)**	11 (28.9%)	9 (3.5%)	**<0.001**

[1] information on bacterial infections and acute kidney injury available in n=287 patients

Table-S10. Cumulative incidences of clinical events in patients with ACLD and low free triiodothyronine (fT3) and non-low fT3 levels

Clinical outcomes	Parameter	0.5 years	1 year	1.5 years	2 years	p-value
**Decompensation/further decompensation**	Low fT3	31.7%	31.7%	31.7%	41.0%	**0.039**
	Non-low fT3	12.1%	16.0%	20.2%	23.9%	
**ACLF**	Low fT3	11.1%	11.1%	17.4%	30.6%	**<0.001**
	Non-low fT3	0.0%	2.8%	4.1%	4.9%	
**Liver-related death**	Low fT3	15.2%	18.6%	18.6%	28.4%	**<0.001**
	Non-low fT3	0.0%	1.8%	1.8%	2.7%	

Table-S11. Impact of low free triiodothyronine (fT3) on the risk of (i) decompensation/further decompensation, (ii) acute-on-chronic liver failure (ACLF) and (iii) liver-related death. Univariate and multivariate multivariate competing risk regression models are shown. Etiological cure, hepatocellular carcinoma, liver transplantation and non-liver-related death were considered as competing risks.

Parameter, Unit	Univariate (unadjusted) analysis			Multivariate (adjusted) analysis		
**(i) Outcome: (further) decompensation**	**sHR**	**95%CI**	**p-value**	**asHR**	**95%CI**	**p-value**
**Low fT3, yes**	1.93	0.99-3.78	0.054	1.33	0.58-3.05	0.510
**Age, 10 years**	0.98	0.81-1.19	0.840	-	-	-
**Sex (male)**	0.90	0.54-1.51	0.690	-	-	-
**Body Mass Index, kg x m^{-2}**	0.99	0.93-1.05	0.720	1.03	0.96-1.11	0.400
**Child Turcotte Pugh score, points**	1.26	1.13-1.39	**<0.001**	1.17	1.01-1.36	**0.041**
**Creatinine, mg x dL^{-1}**	0.83	0.60-1.16	0.280	-	-	-
**Sodium, mmol x L^{-1}**	0.93	0.89-0.98	**0.009**	1.01	0.94-1.08	0.810
**HVPG, mmHg**	1.10	1.07-1.14	**<0.001**	1.09	1.05-1.14	**<0.001**
**C-reactive protein, mg x dL^{-1}**	1.49	1.01-2.19	**0.042**	0.83	0.50-1.37	0.470
**(ii) Outcome: ACLF**	**sHR**	**95%CI**	**p-value**	**asHR**	**95%CI**	**p-value**
**Low fT3, yes**	7.89	3.29-18.90	**<0.001**	4.58	1.77-11.87	**0.002**
**Age, 10 years**	1.27	0.83-1.94	0.270	-	-	-
**Sex (male)**	1.02	0.39-2.65	0.970	-	-	-
**Body Mass Index, kg x m^{-2}**	0.95	0.78-1.15	0.610	1.03	0.95-1.12	0.450
**Child Turcotte Pugh score, points**	1.54	1.28-1.86	**<0.001**	1.26	0.98-1.61	0.071
**Creatinine, mg x dL^{-1}**	1.23	1.06-1.43	**0.007**	0.89	0.71-1.12	0.340
**Sodium, mmol x L^{-1}**	0.85	0.79-0.92	**<0.001**	0.91	0.82-1.01	0.085
**HVPG, mmHg**	1.07	0.92-1.25	0.390	1.09	1.01-1.17	**0.020**

**C-reactive protein, mg x dL^{-1}**	2.21	1.21-4.02	**0.009**	0.91	0.45-1.83	0.780
**(iii) Outcome: Liver-related death**	**sHR**	**95%CI**	**p-value**	**asHR**	**95%CI**	**p-value**
**Low fT3, yes**	12.80	4.55-35.80	**<0.001**	6.80	2.05-22.56	**0.002**
**Age, 10 years**	1.22	0.75-2.01	0.420	-	-	-
**Sex (male)**	0.78	0.27-2.30	0.650	-	-	-
**Body Mass Index, kg x m^{-2}**	0.91	0.79-1.05	0.210	1.04	0.89-1.21	0.640
**Child Turcotte Pugh score, points**	1.63	1.26-2.09	**<0.001**	1.22	0.82-1.80	0.330
**Creatinine, mg x dL^{-1}**	1.03	0.86-1.22	0.780	-	-	-
**Sodium, mmol x L^{-1}**	0.83	0.75-0.91	**<0.001**	0.89	0.76-1.04	0.140
**HVPG, mmHg**	1.15	1.07-1.24	**<0.001**	1.11	1.02-1.21	**0.017**
**C-reactive protein, mg x dL^{-1}**	2.21	1.12-4.35	**0.022**	0.67	0.31-2.12	0.670
