# Supplementary material for: Lower free triiodothyronine (fT3) levels in cirrhosis are linked to systemic inflammation, higher risk of acute-on-chronic liver failure, and mortality
Source: JHEP Rep. 2023 Nov 1;6(1):100954. doi: 10.1016/j.jhepr.2023.100954 (PMC10733101; doi:10.1016/j.jhepr.2023.100954)
Supplement: Multimedia component 4 [file mmc4.pdf]

Lower free triiodothyronine (fT3) levels in cirrhosis are linked to systemic inflammation, higher risk of acute-on-chronic liver failure, and mortality

Lukas Hartl,[1,2] Benedikt Simbrunner,[1,2,3] Mathias Jachs,[1,2] Peter Wolf,[4] David Josef Maria Bauer,[1,2] Bernhard Scheiner,[1,2] Lorenz Balcar,[1,2] Georg Semmler,[1,2] Michael Schwarz,[1] Rodrig Marculescu,[5] Varius Dannenberg,[6] Michael Trauner,[1] Mattias Mandorfer,[1,2] Thomas Reiberger[1,2,3,*]

[1]Division of Gastroenterology and Hepatology, Department of Medicine III, Medical University of Vienna, Vienna, Austria; [2]Vienna Hepatic Hemodynamic Lab, Division of Gastroenterology and Hepatology, Department of Medicine III, Medical University of Vienna, Vienna, Austria; [3]Christian Doppler Lab for Portal Hypertension and Liver Fibrosis, Medical University of Vienna, Vienna, Austria; [4]Division of Endocrinology, Department of Medicine III, Medical University of Vienna, Vienna, Austria; [5]Department of Laboratory Medicine, Medical University of Vienna, Vienna, Austria; [6]Division of Cardiology, Department of Medicine II, Medical University of Vienna, Vienna, Austria

JHEP Reports **2024.** https://doi.org/10.1016/j.jhepr.2023.100954

Background & Aims: Advanced chronic liver disease (ACLD) may affect thyroid hormone homeostasis. We aimed to analyze the pituitary–thyroid axis in ACLD and the prognostic value of free triiodothyronine (fT3).
Methods: Patients with ACLD (liver stiffness measurement [LSM] ≥10 kPa) undergoing hepatic venous pressure gradient (HVPG) measurement between June 2009 and September 2022 and available fT3 levels were included. Clinical stages of ACLD were defined as follows: probable ACLD (pACLD; LSM ≥10 kPa and HVPG ≤5 mmHg), S0 (mild portal hypertension [PH]; HVPG 6–9 mmHg), S1 (clinically significant PH), S2 (clinically significant PH with varices), S3 (past variceal bleeding), S4 (past/current non-bleeding hepatic decompensation), and S5 (further decompensation).
Results: Among 297 patients with ACLD, 129 were compensated (pACLD, n = 10; S0, n = 33; S1, n = 42; S2, n = 44), whereas 168 were decompensated (S3, n = 12; S4, n = 97; S5, n = 59). Median levels of thyroid-stimulating hormone (TSH) numerically increased with progressive ACLD stage (from 1.2 µIU/ml [pACLD] to 1.5 µIU/ml [S5]; p = 0.152), whereas fT3 decreased (from 3.2 pg/ml [pACLD] to 2.5 pg/ml [S5]; p <0.001). Free thyroxin levels remained unchanged (p = 0.338). TSH (aB 0.45; p = 0.046) and fT3 (aB -0.17; p = 0.048) were independently associated with systemic C-reactive protein levels. Lower fT3 was linked to higher risk of (further) decompensation (adjusted subdistribution hazard ratio [asHR] 0.60; 95% CI 0.37–0.97; p = 0.037), acute-on-chronic liver failure (asHR 0.19; 95% CI 0.08–0.49; p <0.001) and liver-related death (asHR 0.14; 95% CI 0.04–0.51; p = 0.003).
Conclusions: Increasing TSH and declining fT3 levels are observed with progressive ACLD stages. The association of TSH and fT3 with systemic inflammation suggests a liver disease-associated non-thyroidal illness syndrome. Lower fT3 levels in patients with ACLD indicate increased risk for decompensation, acute-on-chronic liver failure, and liver-related death.
Impact and Implications: In a large well-characterized cohort of patients with advanced chronic liver disease (ACLD), we found a decline of free triiodothyronine (fT3) throughout the clinical stages of ACLD, paralleled by a numerical increase of thyroid-stimulating hormone (TSH). This suggests a progressive development of a non-thyroidal illness syndrome in association with ACLD severity. Importantly, C-reactive protein independently correlated with TSH and fT3, linking thyroid dysbalance in ACLD to systemic inflammation. Lower fT3 indicated an increased risk for subsequent development of hepatic decompensation, acute-on-chronic liver failure, and liver-related death.
Clinical trial number: Vienna Cirrhosis Study (VICIS; NCT: NCT03267615).
© 2023 The Author(s). Published by Elsevier B.V. on behalf of European Association for the Study of the Liver (EASL). This is an open access article under the CC BY license (http://creativecommons.org/licenses/by/4.0/).

Introduction

Advanced chronic liver disease (ACLD) causes considerable morbidity and mortality worldwide.[1,2] In early asymptomatic stages, ACLD is considered compensated. With the development of portal hypertension (PH)-related complications, including ascites, hepatic encephalopathy (HE), and variceal bleeding, patients progress to decompensated ACLD (dACLD),[3–5] which is

Keywords: Thyroid; TSH; Thyroxin; Triiodothyronine; Inflammation; Advanced chronic liver disease; Portal hypertension; Non-invasive testing.
Received 26 July 2023; received in revised form 2 October 2023; accepted 21 October 2023; available online 1 November 2023
* Corresponding author. Address: Division of Gastroenterology and Hepatology, Department of Internal Medicine III, Waehringer Guertel 18-20, A-1090 Vienna, Austria. Tel.: +4314040065890; Fax: +4314040047350.
E-mail address: thomas.reiberger@meduniwien.ac.at (T. Reiberger).

ELSEVIER
EASL The Home of Hepatology

associated with a considerably worse prognosis.[6,7] Acute-on-chronic liver failure (ACLF), defined by hepatic and/or extrahepatic organ failure(s),[8,9] may occur in patients with dACLD[6,10] and is closely associated with systemic inflammation.[11]

There is a complex interplay between chronic liver disease and thyroid function. On the one hand, the liver is essentially involved in thyroid hormone homeostasis, contributing to their (in) activation, transport, and metabolism.[12] On the other hand, hypothyroidism is known to impair lipid metabolism and promote hepatic steatosis.[13] Accordingly, the risk of non-alcoholic fatty liver disease (NAFLD) increases with lower free thyroxin (fT4) levels.[14] Moreover, increased thyroid-stimulating hormone (TSH) was associated with NAFLD in a meta-analysis.[15] Accordingly, thyroid hormones and thyroid hormone receptor beta agonists are currently evaluated as potential therapies for NAFLD.[16]

Furthermore, a small study including 33 patients with liver cirrhosis reported lower levels of triiodothyronine (T3) and free T3 (fT3), as well as lower T3/thyroxin-binding globulin (TBG) and thyroxin (T4)/TBG ratios.[17] A typical altered pattern of thyroid hormone homeostasis with decreased levels of fT3 and similar levels of fT4 was described in cirrhosis, and defined as ‘low fT3 syndrome’.[18] Moreover, a small retrospective study demonstrated shorter survival among patients with low fT3,[19] linking dysregulated thyroid signaling to impaired clinical outcomes in patients with cirrhosis.

However, abnormalities of the pituitary–thyroid axis across the distinct substages of cirrhosis, as well as the impact of fT3 on different clinical outcomes, have not yet been systematically investigated. Thus, this study aimed (i) to investigate the pituitary–thyroid axis throughout the individual clinical stages of ACLD,[6] (ii) to analyze parameters associated with the dysregulation of thyroid hormones in ACLD, and (iii) to determine the impact of fT3 on clinical outcomes of ACLD.

Patients and methods

Study design

This study investigated consecutive patients with suspected ACLD (*i.e.* liver stiffness measurement [LSM] ≥10 kPa) undergoing HVPG measurement at the Hepatic Hemodynamic Lab of the Vienna General Hospital between June 2009 and September 2022 following a standardized operating procedure.[20] We exclusively included clinically stable patients in whom data on TSH and fT3 were available.

Patients with a history of liver transplantation (LT), hepatocellular carcinoma (HCC) or other malignancy, vascular liver disease, portal vein thrombosis (PVT), transjugular intrahepatic portosystemic shunt (TIPS), congestive heart disease, intake of thyroid hormones, or active infection at the time of HVPG measurement were excluded. Moreover, we excluded patients with insufficient available clinical or laboratory data.

Central venous blood withdrawals for assessment of pituitary–thyroid axis parameters and HVPG measurements were conducted under fasting conditions. Clinical events occurring during follow-up (FU) including the development of decompensation events (clinically apparent ascites, variceal bleeding, HE, and ACLF), as well as acute kidney injury, HCC, LT, and (liver-related) death, were documented.

Patient cohorts

Patients were stratified for ACLD severity according to clinical stages modified from D’Amico *et al.*[6] for baseline characterization of the pituitary–thyroid axis in patients with ACLD. Compensated ACLD (cACLD) was defined by the absence of any previous decompensating event defined and was further subdivided into four distinct stages: probable ACLD (pACLD; LSM ≥10 kPa and HVPG ≤5 mmHg),[21] S0 (mild PH; HVPG 6–9 mmHg), S1 (clinically significant PH [*i.e.* HVPG ≥10 mmHg] without the presence of varices), and S2 (clinically significant PH with the presence of varices). dACLD was constituted by a history of at least one decompensation event. Patients with dACLD were assigned to one of three substages: S3 (history of acute variceal bleeding), S4 (history of decompensation as a result of non-bleeding decompensating events including ascites and HE), and S5 (further decompensation; *i.e.* history of two or more different types of decompensating events).

Decompensation events

In cACLD, decompensation was defined by occurrence of clinically evident ascites, HE, or variceal bleeding. In dACLD, further decompensation was defined by either occurrence of a second type of hepatic decompensation or worsening of the first decompensation. Worsening included development of spontaneous bacterial peritonitis (SBP) or refractory ascites in patients with ascites, first hospital (re-) admission caused by HE in patients with previous HE, and variceal rebleeding in patients with a history of variceal bleeding. Finally, ACLF following the EASL/EF-CLIF definition[10,22] was regarded as a fourth and distinct type of decompensating event, which could potentially occur in patients with cACLD and those with dACLD.

Assessment of HVPG and LSM

A standardized HVPG measurement procedure was followed as previously described.[20] In brief, a catheter introducer sheath was placed in the right internal jugular vein following local anesthesia. Subsequently, a specifically designed angled-tip balloon catheter[23] was inserted into a large hepatic vein under fluoroscopic guidance. HVPG was calculated by subtracting free from wedged hepatic vein pressure. A total of three measurements were performed per patient, and the median HVPG of these measurements was used for further analyses. Vibration-controlled transient elastography (FibroScan®; Echosens, Paris, France) under fasting conditions was used for LSM, as previously described.[24]

Pituitary–thyroid axis parameters and routine laboratory parameters

All laboratory tests were conducted at the ISO-certified Department of Laboratory Medicine of the Vienna General Hospital. Blood withdrawals were conducted in a standardized manner after HVPG measurement under fasting conditions and after resting in the supine position for at least 30 min. Serum levels of TSH (normal range 0.27–4.2 µIU/ml; Elecsys TSH, Roche Diagnostics, Mannheim, Germany), as well as fT4 (normal range 0.76–1.66 ng/dl; Elecsys fT4 III, Roche Diagnostics, Mannheim, Germany) and fT3 (normal range 2.15–4.12 pg/ml; Elecsys fT3 III, Roche Diagnostics) were analyzed by electrochemoluminescence immunoassay (Roche Diagnostics). Standard laboratory methods were used for the assessment of routine laboratory parameters. Low fT4 and low fT3 were defined as fT4 <lower limit of normal (LLN) and fT3 <LLN, respectively. Elevated TSH was defined as TSH >upper limit of normal (ULN).

Statistical analysis

The number (n) and proportion (%) of patients with the parameter of interest were reported for categorical variables. Continuous data were presented using median and IQR. For comparison of continuous non-normally distributed variables between two groups, the Mann–Whitney *U* test was implemented. The Kruskal–Wallis test was performed for comparing continuous variables in three or more groups. Pearson's Chi-square test or Fisher's exact test, as appropriate, was used for group comparisons of categorical variables. Spearman's rho (ρ) was used to assess correlations. Linear regression analysis was conducted to investigate factors associated with TSH and fT3. Clinical outcomes at half-year intervals up to 2 years of FU were assessed using cumulative incidence calculation. Gray's test, as previously described,[25] was computed for cumulative incidence comparison. Fine and Gray competing risk regression models using the R package cmprsk[25,26] were calculated to evaluate whether TSH, fT4, and fT3 levels were associated with the risk of clinical events of interest. Apart from TSH, fT4, and fT3, well-established risk factors for worse outcomes in ACLD (*i.e.* age, Child–Turcotte–Pugh [CTP] score, serum creatinine, sodium, HVPG as a marker for PH, and C-reactive protein [CRP] as a parameter of systemic inflammation) as well as sex were evaluated by univariate and multivariate analysis. Etiological cure, HCC, LT, and non-liver-related death were considered as competing risks for both cumulative incidence comparisons and competing risk regression models. In both the linear regression models and the competing risk regression models, parameters that were associated with the parameter of interest in univariate analysis (p <0.100) were included in the multivariate model. Moreover, TSH, fT3, or fT4 as the main target parameters, as well as BMI, CTP score, and HVPG as established parameters of pathophysiological and prognostic relevance, were included in the multivariate models. IBM SPSS 27.0 statistic software (IBM, Armonk, NY, USA), R 4.2.1 (R Core Team, R Foundation for Statistical Computing, Vienna, Austria), and GraphPad Prism 8 (GraphPad Software, La Jolla, CA, USA) were used for statistical analyses. A two-sided p value of <0.05 was considered statistically significant.

Ethics

The study was approved by the ethics committee of the Medical University of Vienna (No. 1493/2016 and No. 1262/2017) and was performed in accordance with the current version of the Helsinki Declaration. All patients included between 2018 and 2022 are part of the prospective Vienna Cirrhosis Study (VICIS; NCT: NCT03267615) and gave their written informed consent before study inclusion. The ethics committee waved the need for informed consent for the retrospective part of the study.

Results

Patient characteristics

In total, 297 patients with ACLD with a median age of 56.0 years and male predominance (67.0%) were included in the study. The patient flow chart is depicted in Fig. 1. Baseline characteristics of the included patients are provided in Table 1. The main etiologies were alcohol-related liver disease (53.9%), viral hepatitis (16.5%), and metabolic dysfunction-associated steatohepatitis (MASH; 11.8%). Overall, the study included 56.6% of patients with dACLD – most frequently caused by ascites (85.7% of patients with dACLD). The median HVPG was 17 mmHg, and the median model for end-stage liver disease (MELD) was 11 points.

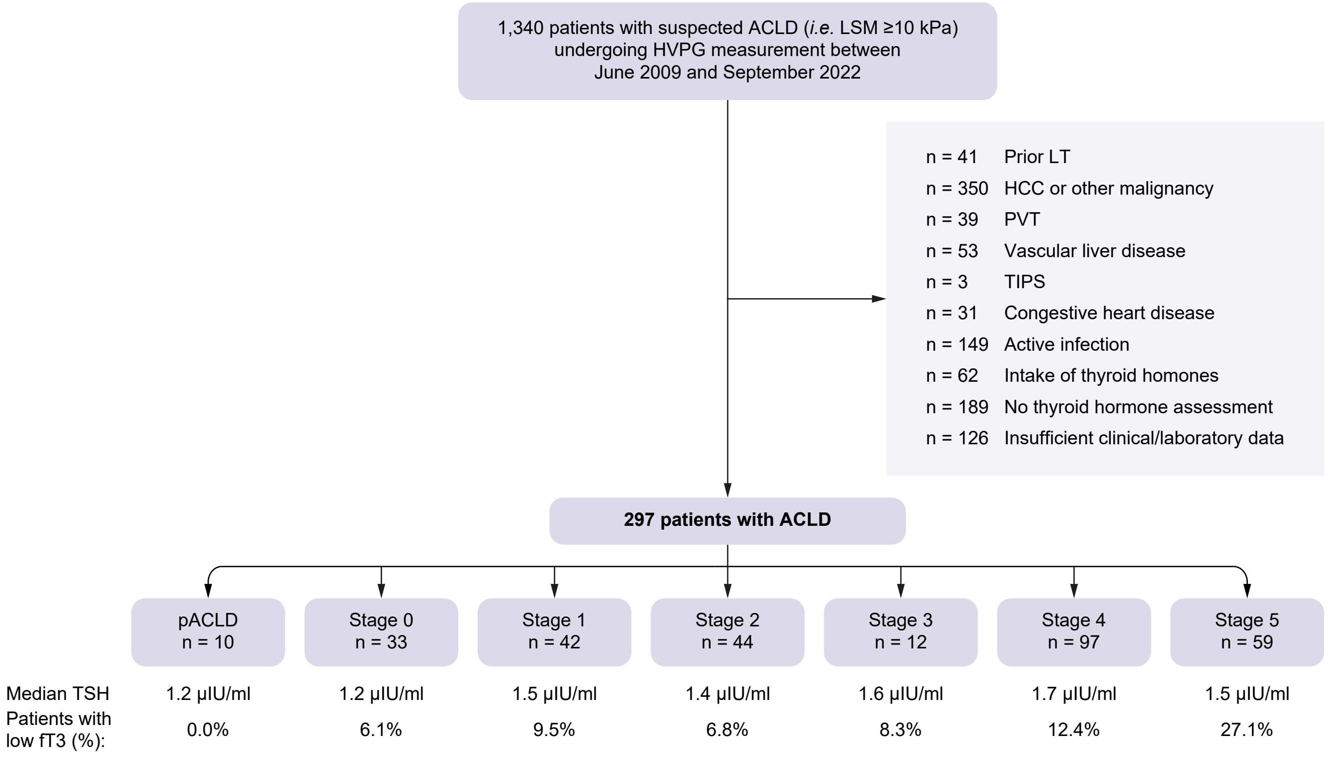

Fig. 1. Patient flow chart. ACLD, advanced chronic liver disease; fT3, free triiodothyronine; HCC, hepatocellular carcinoma; HVPG, hepatic venous pressure gradient; LSM, liver stiffness measurement; LT, liver transplantation; pACLD, probable ACLD (*i.e.* LSM ≥10 kPa and HVPG ≤5 mmHg); PVT, portal vein thrombosis; TIPS, transjugular intrahepatic portosystemic shunt; TSH, thyroid-stimulating hormone.

Table 1. Patient characteristics.

Patient characteristics	All patients (N = 297)
Sex, male/female (% male)	199/98 (67.0)
Age (years), median (IQR)	56.0 (48.0–63.8)
BMI (kg/m^2), median (IQR)	26.0 (22.9-29.7)
Etiology	
ALD, n (%)	160 (53.9)
Viral hepatitis, n (%)	49 (16.5)
MASH, n (%)	35 (11.8)
Cholestatic, n (%)	21 (7.1)
AIH, n (%)	9 (3.0)
Other, n (%)	23 (7.7)
Decompensated ACLD, n (%)	168 (56.6)
Ascites, n (%)	144 (48.5)
Refractory ascites, n (%)	27 (9.1)
Hepatic encephalopathy, n (%)	54 (18.2)
Variceal bleeding, n (%)	35 (11.8)
EASL stage	
Probable ACLD, n (%)	10 (3.4)
0, n (%)	33 (11.1)
1, n (%)	42 (14.1)
2, n (%)	44 (14.8)
3, n (%)	12 (4.0)
4, n (%)	97 (32.7)
5, n (%)	59 (19.9)
MELD (points), median (IQR)	11 (9–15)
CTP score (points), median (IQR)	6 (5–8)
CTP stage	
A, n (%)	159 (53.5)
B, n (%)	102 (34.3)
C, n (%)	36 (12.1)
Bilirubin (mg/dl), median (IQR)	1.1 (0.7–2.3)
Albumin (g/dl), median (IQR)	36.9 (32.8–40.2)
INR (U), median (IQR)	1.4 (1.2–1.6)
Sodium (mmol/L), median (IQR)	138.0 (136.0–140.5)
HVPG (mmHg), median (IQR)	17 (11–20)
MAP (mmHg), median (IQR)	99 (90–111)
LSM (kPa), median (IQR)	36.5 (22.0–61.7)
TSH (µIU/ml), median (IQR)	1.5 (1.1–2.2)
fT3 (pg/ml), median (IQR)	2.8 (2.4–3.2)
fT4 (ng/dl), median (IQR)	1.2 (1.0–1.3)
IL-6* (ng/dl), median (IQR)	8.4 (4.7–15.7)
WBC (G/L), median (IQR)	4.8 (3.5–6.3)
CRP (mg/dl), median (IQR)	0.2 (0.1–0.6)

ACLD, advanced chronic liver disease; AIH, autoimmune hepatitis; ALD, alcohol-related liver disease; CRP, C-reactive protein; CTP, Child–Turcotte–Pugh; fT3, free triiodothyronine; fT4, free thyroxin; HVPG, hepatic venous pressure gradient; INR, international normalized ratio; LSM, liver stiffness measurement; MAP, mean arterial pressure; MASH, metabolic dysfunction-associated steatohepatitis; MELD, model for end-stage liver disease; TSH, thyroid-stimulating hormone; WBC, white blood cell count.
* Available in 286 patients.

Pituitary–thyroid axis parameters in patients with different clinical stages of ACLD

With progressive ACLD severity, patients exhibited numerically higher levels of TSH (from 1.2 µIU/ml [pACLD] to 1.5 µIU/ml [S5]; p = 0.152; Fig. 2 and Table S1). Still, TSH mostly remained within the normal range with only overall nine patients (3.0%; no difference between distinct clinical stages; see Table S1) exhibiting elevated levels. At the same time, median fT3 levels decreased throughout the clinical substages (from 3.2 pg/ml [pACLD] to 2.5 pg/ml [S5]; p <0.001), whereas median fT4 remained unchanged (1.2 ng/dl [pACLD] *vs.* 1.2 ng/dl [S5]; p = 0.338). Importantly, 12.8% (n = 38/297) of patients with ACLD had low (*i.e.* <LLN) fT3 levels with significantly higher prevalence among advanced clinical stages (0.0% [pACLD] *vs.* 27.1% [S5]; p = 0.015). Table 2 depicts the levels of parameters of the pituitary–thyroid axis in cACLD compared with dACLD.

Moreover, IL-6 (from 4.6 ng/dl [pACLD] to 13.5 ng/dl [S5]; p <0.001) and CRP (from 0.2 mg/dl [pACLD] to 0.4 mg/dl [S5]; p <0.001), as well as parameters of hepatic dysfunction (see Table S1), significantly increased with progressive clinical ACLD stage.

Interestingly, as detailed in Table S2, TSH and fT3 did not differ between distinct BMI strata in both patients with cACLD and patients with dACLD.

Correlations of parameters of the pituitary–adrenal axis, liver function, and systemic inflammation

Levels fT3 did not correlate with fT4 (ρ = 0.000; p = 0.998; Fig. 3) and only weakly correlated with TSH (ρ = -0.152; p = 0.009). Similarly, fT4 showed no correlation with TSH (ρ = -0.109; p = 0.061). Interestingly, there were moderate correlations between fT3 and parameters of liver dysfunction (MELD: ρ = -0.448; p <0.001; and albumin: ρ = 0.434; p <0.001) and systemic inflammation (IL-6: ρ = -0.496; p <0.001; and CRP: ρ = -0.356; p <0.001). Furthermore, fT3 correlated with parameters of PH (HVPG: ρ = -0.300; p <0.001), endothelial dysfunction (von Willebrand factor antigen [VWF]: ρ = -0.357; p <0.001), and hyperdynamic circulation (pro-brain-type natriuretic peptide [proBNP]: ρ = -0.385; p <0.001). By contrast, fT4 and TSH showed no or merely weak correlations with these parameters (see Fig. 3 and Table S3).

In multivariate linear regression analysis, fT3 was independently linked to CRP (per mg/dl; aB -0.17; p = 0.048; Table S4), CTP score (per point; aB -0.15; p <0.001), and age (per 10 years; aB -0.11; p <0.001), whereas the only parameter independently associated with TSH was CRP (per mg/dl; aB 0.45; p = 0.046). In addition, we demonstrated a significant relation of fT3 and systemic inflammation within the subgroup of patients with dACLD on linear regression analysis (Table S5).

FU and clinical outcomes

The median FU period was 419.0 days. Overall, 25.3% of patients (n = 75) experienced at least one (further) decompensation event. Details on the rates of different decompensation events are given in Table 3. Ten patients (3.4%) developed HCC during FU. TIPS implantation was conducted in 5.7% (n = 17) of patients, and 19 patients (6.4%) underwent LT. Furthermore, 11.1% (n = 33) of patients died with 20 of these deaths (60.6%) being attributed to liver disease.

Impact of TSH, fT4, and fT3 on clinical outcomes in patients with ACLD

Table 4 shows the impact of fT3 on the risk of (further) decompensation, ACLF, and liver-related death. After adjustment for relevant cofactors, lower fT3 was associated with higher risk of (further) decompensation (adjusted subdistribution hazard ratio [asHR] 0.60; 95% CI 0.37–0.97; p = 0.037), ACLF (asHR 0.19; 95% CI 0.08–0.49; p <0.001), and liver-related death (asHR 0.14; 95% CI 0.04–0.51; p = 0.003). TSH (see Table S6) and fT4 (see Table S7) were not linked to adverse outcomes in multivariate analyses.

Low fT3 for risk stratification in patients with ACLD

A detailed comparison of characteristics of patients with and without low fT3 is provided in Table S8. Patients with low fT3 more frequently developed (further) decompensation (36.8% *vs.* 23.6% [non-low fT3]; p = 0.078; Table S9), ACLF (28.9% *vs.* 5.8% [non-low fT3]; p <0.001), and liver-related death (28.9% *vs.* 3.5%

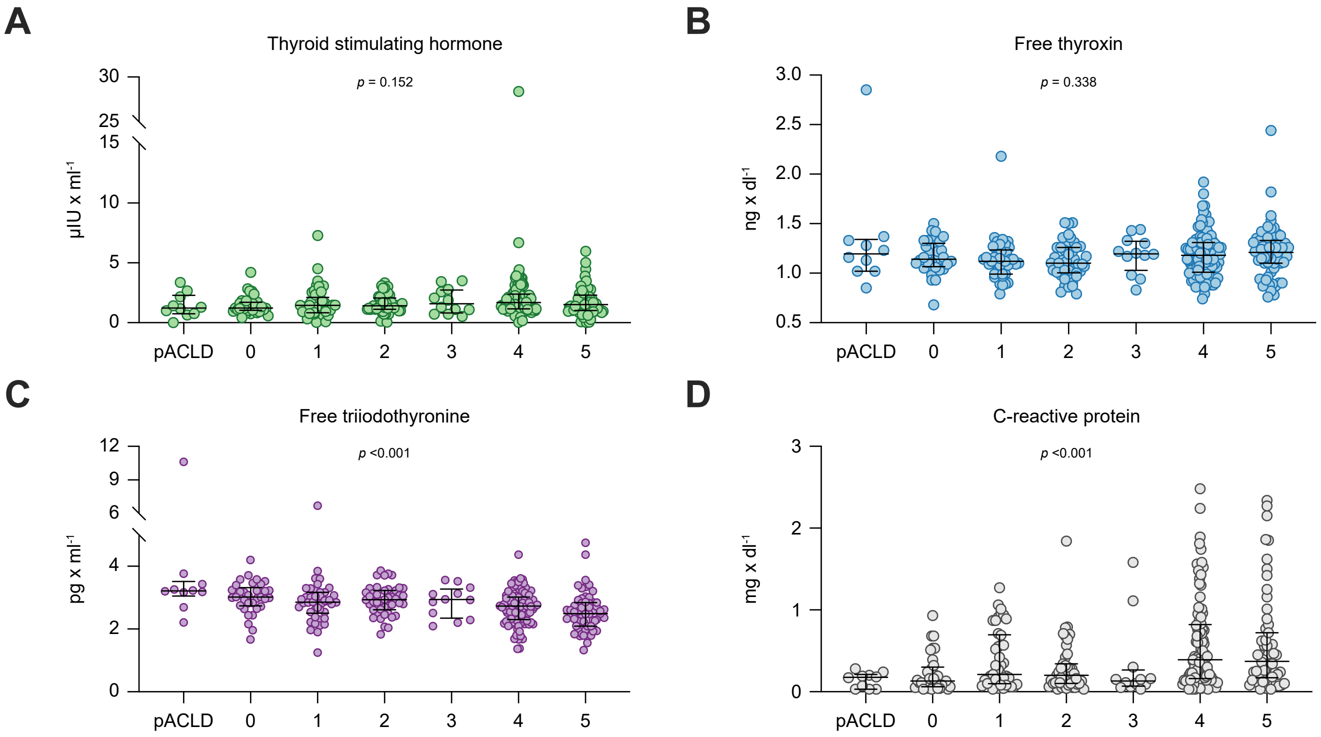

Fig. 2. Plasma levels of thyroid-stimulating hormone, free thyroxin, free triiodothyronine, and C-reactive protein in patients with different stages of ACLD. Statistical differences between the groups were assessed using Kruskal-Wallis test. Patients were stratified by EASL stages with the addition of patients with pACLD (*i.e.* HVPG ≤5 mmHg and LSM ≥10 kPa). HVPG, hepatic venous pressure gradient; LSM, liver stiffness measurement; pACLD, probable advanced chronic liver disease.

Table 2. Levels of parameters of the pituitary–thyroid axis, liver function, and systemic inflammation in patients with cACLD and dACLD.

Parameter	cACLD (n = 129), median (IQR)	dACLD (n = 168), median (IQR)	*p* value
TSH (µIU/ml)	1.4 (1.0–1.9)	1.6 (1.1–2.4)	0.012
fT3 (pg/ml)	3.0 (2.6–3.2)	2.7 (2.3–3.0)	<0.001
fT4 (ng/dl)	1.1 (1.0–1.3)	1.2 (1.0–1.3)	0.006
CTP score (points)	5.0 (5.0–6.0)	7.0 (6.0–9.0)	<0.001
MELD (points)	9.0 (8.0–13.0)	14.0 (10.0–17.0)	<0.001
Bilirubin (mg/dl)	0.9 (0.7–1.8)	1.5 (0.8–2.6)	<0.001
Albumin (g/dl)	39.0 (36.1–41.8)	35.3 (31.1–38.7)	<0.001
IL-6* (ng/dl)	5.6 (3.4–9.5)	11.5 (7.1–20.7)	<0.001
CRP (mg/dl)	0.2 (0.1–0.4)	0.4 (0.1–0.8)	<0.001

The groups were compared via Mann-Whitney *U* test. cACLD, compensated advanced chronic liver disease; CRP, C-reactive protein; CTP, Child–Turcotte–Pugh; dACLD, decompensated advanced chronic liver disease; fT3, free triiodothyronine; fT4, free thyroxin; MELD, model for end-stage liver disease; TSH, thyroid stimulating hormone.
* Available in 286 patients (cACLD, n = 124; dACLD, n = 162).

[non-low fT3]; *p* <0.001). Moreover, the rate of SBP was higher in the low fT3 group (7.9% *vs.* 1.9% [non-low fT3]; *p* = 0.034).

Patients with low fT3 exhibited a higher cumulative incidence of (further) decompensation (*p* = 0.039; Table S10), ACLF (*p* <0.001), and liver-related death (*p* <0.001) (Fig. 4). The cumulative incidence of liver-related death was significantly higher among patients with low fT3 both at 1 year (18.6% [low fT3] *vs.* 1.8% [non-low fT3]) and at 2 years of FU (28.4% [low fT3] *vs.* 2.7% [non-low fT3]; *p* <0.001).

After adjustment for clinically relevant cofactors, low fT3 was independently associated with higher risk of ACLF (asHR 4.58; 95% CI 1.77–11.87; *p* = 0.002; Table S11) and liver-related death (asHR 6.80; 95% CI 2.05–22.56; *p* = 0.002). In multivariate analysis, low fT3 was not linked to (further) decompensation (asHR 1.33; 95% CI 0.58–3.05; *p* = 0.510).

Discussion

In this study, we demonstrated that the pituitary–thyroid signaling axis becomes gradually impaired with progressing stages of ACLD and that low fT3 levels are of critical prognostic value in a large well-characterized cohort of patients with ACLD. Specifically, lower fT3 levels across the distinct clinical ACLD substages were paralleled by increases in TSH, whereas fT4 remains mostly unchanged. Importantly, fT3 and TSH were independently linked to CRP, suggesting a key role of systemic inflammation in the dysregulation of thyroid hormone signaling in ACLD. Furthermore, lower fT3 levels correlated with parameters of liver dysfunction, PH, endothelial dysfunction, and hyperdynamic circulation, indicating a potential role of altered thyroid signaling in ACLD progression. Finally, we demonstrated that fT3 – as both a continuous variable and dichotomized

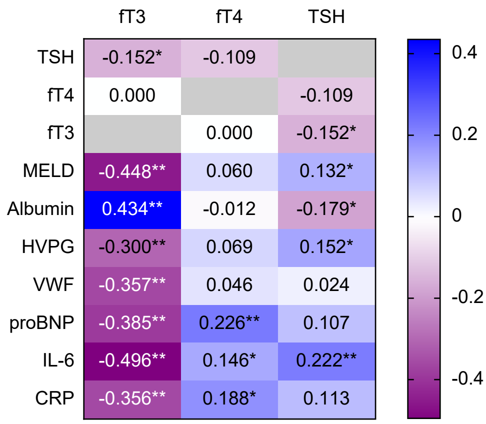

Fig. 3. Correlations of fT3, fT4, and TSH with parameters of the pituitary–thyroid axis, liver dysfunction, portal hypertension, endothelial dysfunction, hyperdynamic circulation, and systemic inflammation. Correlations are indicated as Spearman's correlation coefficient. Asterisks signify significant correlations: **p* <0.050; ***p* <0.001. CRP, C-reactive protein; fT3, free triiodothyronine; fT4, free thyroxin; HVPG, hepatic venous pressure gradient; MELD, model for end-stage liver disease; proBNP, pro-brain-type natriuretic peptide; TSH, thyroid-stimulating hormone; VWF, von Willebrand factor antigen.

Table 3. Follow-up, TIPS implantation, and clinical outcomes.

Clinical outcome	All patients (N = 297)
Follow-up time (days), median (IQR)	419.0 (156.5–797.5)
Decompensation event, n (%)	75 (25.3)
Ascitic complication, n (%)	43 (14.5)
Variceal bleeding, n (%)	13 (4.4)
Hepatic encephalopathy, n (%)	39 (13.1)
ACLF, n (%)	26 (8.8)
Bacterial infections*, n (%)	38 (12.8)
SBP, n (%)	8 (2.7)
Acute kidney injury*, n (%)	24 (8.1)
HCC, n (%)	10 (3.4)
TIPS implantation, n (%)	17 (5.7)
Liver transplantation, n (%)	19 (6.4)
Death, n (%)	33 (11.1)
Liver-related death, n (%)	20 (6.7)

ACLF, acute-on-chronic liver failure; HCC, hepatocellular carcinoma; SBP, spontaneous bacterial peritonitis; TIPS, transjugular intrahepatic portosystemic shunt.
* Information on bacterial infections and acute kidney injury is available in n = 287 patients.

variable – confers important prognostic value, as it was an independent predictor of ACLF development and liver-related mortality.

It has long been known that thyroid hormone dysregulation occurs in liver cirrhosis[17] with abnormalities of thyroid hormones described in 13–61% of patients.[12] Typically, patients with ACLD exhibit decreased total T3, fT3, and fT4, as well as increased reverse T3 compared with healthy individuals, while usually maintaining normal levels of TSH.[12,27,28] Accordingly, in our study the majority of patients with ACLD showed euthyroid function. Furthermore, previous studies have shown that lower fT3 is associated with hepatic dysfunction (*i.e.* higher Child–Pugh scores),[12,27] whereas there were no differences in the levels of fT4 between patients with compensated and decompensated cirrhosis.[27,28]

This is in line with our data, demonstrating that fT3 significantly declines with progressive clinical stage of ACLD, along with increasing TSH levels with progressive clinical ACLD stage. Interestingly, the prevalence of elevated TSH was low (3.0% of patients) and did not differ between different stages of ACLD, and median levels of fT4 were virtually identical in different substages of ACLD, indicating that patients with dACLD, despite exhibiting progressively lower fT3 seem to mostly remain in a euthyroid state.

These thyroid hormone alterations correspond to a non-thyroidal illness syndrome or euthyroid sick syndrome, which can be described to occur in patients with severe or chronic illness.[29,30] Different underlying pathogenetic mechanisms have been proposed, including a downregulation of type 1 deiodinase, hampering the conversion of T4 to T3, reduced hepatic clearance of reverse T3, or changes in the concentration of thyroid hormone binding proteins in patients with ACLD.[12] Notably, we demonstrate a strong linear correlation between lower fT3 and higher CRP, which strongly suggests a critical contribution of systemic inflammation to lowered fT3 levels with progressive ACLD severity.

Generally, patients with ACLD – particularly those with dACLD – exhibit increased levels of systemic inflammation[5] that is mechanistically caused by intestinal dysbiosis and bacterial translocation.[31–33] It has been demonstrated that chronic inflammation leads to altered transport and metabolism of thyroid hormones.[29,34] Moreover, systemic inflammation has also

Table 4. Impact of fT3 on the risk of (i) decompensation or further decompensation, (ii) ACLF, and (iii) liver-related death.

	Univariate (unadjusted) analysis			Multivariate (adjusted) analysis		
**Parameter**	**sHR**	**95% CI**	***p* value**	**asHR**	**95% CI**	***p* value**
**(i) Outcome: (further) decompensation**						
fT3 (pg/ml)	0.48	0.32–0.72	**<0.001**	0.60	0.37–0.97	**0.037**
Age (10 years)	0.98	0.81–1.19	0.840	–	–	–
Sex (male)	0.90	0.54–1.51	0.690	–	–	–
BMI (kg/m^2)	0.99	0.93–1.05	0.720	1.03	0.96–1.10	0.400
Child–Turcotte–Pugh score (points)	1.26	1.13–1.39	**<0.001**	1.12	0.96–1.30	0.160
Creatinine (mg/dl)	0.83	0.60–1.16	0.280	–	–	–
Sodium (mmol/L)	0.93	0.89–0.98	**0.009**	1.01	0.94–1.09	0.800
HVPG (mmHg)	1.10	1.07–1.14	**<0.001**	1.09	1.05–1.14	**<0.001**
C-reactive protein (mg/dl)	1.49	1.01–2.19	**0.042**	0.78	0.47–1.30	0.340
**(ii) Outcome: ACLF**						
fT3 (pg/ml)	0.15	0.07–0.33	**<0.001**	0.19	0.08–0.49	**<0.001**
Age (10 years)	1.27	0.83–1.94	0.270	–	–	–
Sex (male)	1.02	0.39–2.65	0.970	–	–	–
BMI (kg/m^2)	0.95	0.78–1.15	0.610	1.02	0.94–1.11	0.630
Child–Turcotte–Pugh score (points)	1.54	1.28–1.86	**<0.001**	1.16	0.88–1.52	0.300

(*continued on next page*)

Table 4 (*continued*)

	Univariate (unadjusted) analysis			Multivariate (adjusted) analysis		
**Parameter**	**sHR**	**95% CI**	***p* value**	**asHR**	**95% CI**	***p* value**
Creatinine (mg/dl)	1.23	1.06–1.43	**0.007**	0.87	0.68–1.12	0.290
Sodium (mmol/L)	0.85	0.79–0.92	**<0.001**	0.90	0.79–1.01	0.077
HVPG (mmHg)	1.07	0.92–1.25	0.390	1.11	1.02–1.20	**0.011**
C-reactive protein (mg/dl)	2.21	1.21–4.02	**0.009**	0.82	0.39–1.73	0.610
**(iii) Outcome: Liver-related death**						
fT3 (pg/ml)	0.11	0.04–0.30	**<0.001**	0.14	0.04–0.51	**0.003**
Age (10 years)	1.22	0.75–2.01	0.420	–	–	–
Sex (male)	0.78	0.27–2.30	0.650	–	–	–
BMI (kg/m^2)	0.91	0.79–1.05	0.210	1.02	0.88–1.18	0.780
Child–Turcotte–Pugh score (points)	1.63	1.26–2.09	**<0.001**	1.12	0.75–1.66	0.580
Creatinine (mg/dl)	1.03	0.86–1.22	0.780	–	–	–
Sodium (mmol/L)	0.83	0.75–0.91	**<0.001**	0.89	0.75–1.05	0.150
HVPG (mmHg)	1.15	1.07–1.24	**<0.001**	1.13	1.03–1.25	**0.013**
C-reactive protein (mg/dl)	2.21	1.12–4.35	**0.022**	0.75	0.28–2.02	0.570

Univariate and multivariate Fine and Grey competing risk regression models are shown. Etiological cure, hepatocellular carcinoma, liver transplantation, and non-liver-related death were considered as competing risks. *P* values in bold denote statistical significance.
ACLF, acute-on-chronic liver failure; asHR, adjusted sHR; fT3, free triiodothyronine; HVPG, hepatic venous pressure gradient; sHR, subdistribution hazard ratio.

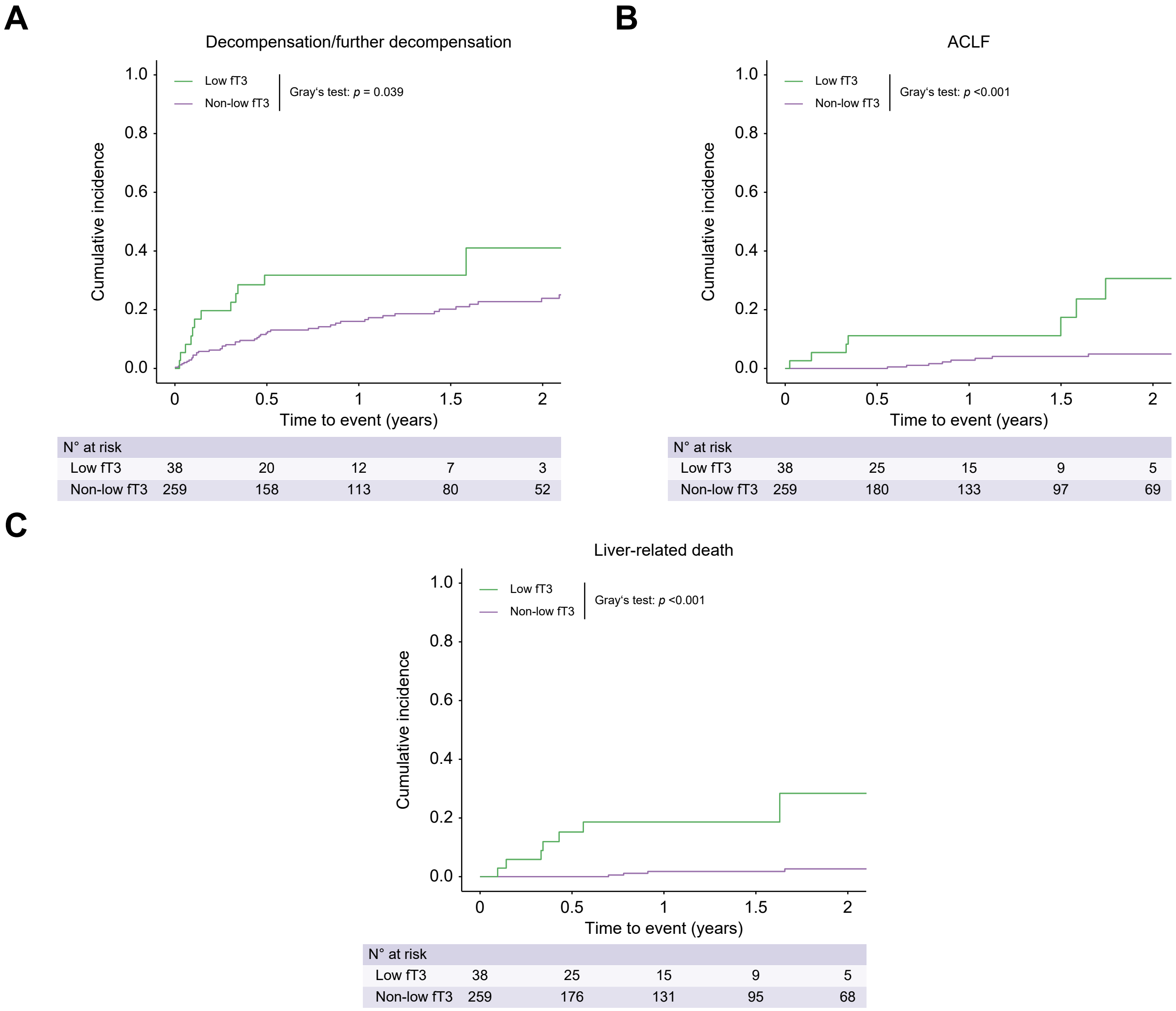

Fig. 4. Cumulative incidence of first/further hepatic decompensation, ACLF, and liver-related death within 2 years of follow-up stratified by fT3 levels. Etiological cure, hepatocellular carcinoma, liver transplantation, and non-liver-related death were considered as competing risks. Cumulative incidences were compared using Gray's test. ACLF, acute-on-chronic liver failure; fT3, free triiodothyronine.

been linked to a suppression of the pituitary–adrenal axis in ACLD,[35] and indeed, CRP was the only parameter independently linked to TSH in our study population. Thus, chronic inflammation in patients with advanced stages of ACLD may contribute to the dysregulation of thyroid hormone homeostasis both by impairment of the pituitary–thyroid axis and by influencing hormone transport and metabolism.

Although a detrimental effect of low fT3 (*i.e.* fT3 <LLN) on survival in patients with cirrhosis has been previously described,[19] our study adds to this finding by demonstrating that lower fT3 levels – even when remaining in the normal range – when considering them as continuous, and not only as a binary parameter, are strongly linked to development of ACLF and liver-related death. On univariate analyses, lower fT3 levels also predicted (further) decompensation in patients with ACLD, whereas TSH and fT4 did not show any correlations with clinical outcomes. Mechanistically, fT3 showed a correlation to parameters of hepatic dysfunction (*i.e.* MELD and albumin), PH (*i.e.* HVPG), endothelial dysfunction (*i.e.* VWF[36,37]), and hyperdynamic circulation (*i.e.* proBNP[7]), suggesting a role of fT3 in ACLD progression. At this point, further mechanistic studies are required to decipher the impact of fT3 hormones on ACLD progression and particularly on immune dysfunction (cirrhosis-associated immune dysfunction).

Our study also has limitations. Firstly, we did not assess levels of thyroglobulin or reverse T3 and can thus not comment on their role in altered thyroid hormone signaling in ACLD. Thyroglobulin and reverse T3 were previously reported to be elevated in liver cirrhosis.[12,38] Secondly, the retrospective design and the long study period represent another limitation. Importantly, we started to systematically assess fT4 and fT3 in April 2018 in patients who were prospectively included and followed up in the VICIS study, which includes 96.4% (n = 287/297) of patients in this study. By assessing thyroid hormones in consecutive patients undergoing HVPG measurement, we can largely exclude selection bias. Thirdly, we could not measure intrahepatic deiodinase expression/activity and systemic T3/T4-binding globulin levels to assess potential loco-hepatic hypothyroidism and dysregulation of the thyroid hormone metabolism in ACLD, respectively. Moreover, we did not assess anti-thyroglobulin or anti-thyroid peroxidase antibodies. However, no patient with autoimmune thyroid disease or intake of antithyroid medication was included in our study. Furthermore, only 11.8% of patients included in this study had MASH as primary liver disease etiology. Further research is required to explore the specific impact of thyroid hormone imbalance in patients with metabolic dysfunction-associated steatotic liver disease and MASH. Finally, we did not assess nutritional status or sarcopenia, which may influence fT3 levels.

In conclusion, our large observational study showed a significant increase in TSH and decline of fT3 levels with progressive clinical stage of ACLD. Mechanistically, lower fT3 seems to be linked to liver dysfunction, PH, endothelial dysfunction, and hyperdynamic circulation. Interestingly, both lower fT3 and higher TSH were associated with biomarkers of systemic inflammation.

Finally, and clinically most relevant, lower fT3 levels were independent predictors of hepatic decompensation, ACLF, and liver-related mortality. Based on our study results, regular monitoring of thyroid hormones – particularly fT3 – in patients with ACLD is warranted. Further studies are required to determine whether specific therapeutic interventions correcting thyroid hormone imbalances may benefit patients with ACLD.

Abbreviations

ACLD, advanced chronic liver disease; ACLF, acute-on-chronic liver failure; asHR, adjusted sHR; AIH, autoimmune hepatitis; ALD, alcohol-related liver disease; cACLD, compensated ACLD; CRP, C-reactive protein; CTP, Child–Turcotte–Pugh; dACLD, decompensated ACLD; fT3, free triiodothyronine; fT4, free thyroxin; FU, follow-up; HCC, hepatocellular carcinoma; HE, hepatic encephalopathy; HVPG, hepatic venous pressure gradient; INR, international normalized ratio; LLN, lower limit of normal; LSM, liver stiffness measurement; LT, liver transplantation; MAP, mean arterial pressure; MASH, metabolic dysfunction-associated steatohepatitis; MELD, model for end-stage liver disease; pACLD, probable ACLD; PH, portal hypertension; proBNP, pro-brain-type natriuretic peptide; PVT, portal vein thrombosis; SBP, spontaneous bacterial peritonitis; sHR, subdistribution hazard ratio; T3, triiodothyronine; T4, thyroxin; TBG, thyroxin-binding globulin; TIPS, transjugular intrahepatic portosystemic shunt; TSH, thyroid-stimulating hormone; ULN, upper limit of normal; VICIS, Vienna Cirrhosis Study; VWF, von Willebrand factor antigen; WBC, white blood cell count.

Financial support

No specific funding was received for this study.

Conflicts of interest

The authors have nothing to disclose regarding the work under consideration for publication. Conflicts of interest outside the submitted work: LH, MJ, PW, LB, GS, MS, RM, and VD have nothing to disclose. BSim received travel support from AbbVie and Gilead. DJMB received speaker fees from AbbVie and Siemens, grant support form Gilead and Siemens, and travel support from AbbVie and Gilead. BSch received travel support from AbbVie, Ipsen, and Gilead. MT served as a speaker and/or consultant and/or advisory board member for Albireo, BiomX, Falk, Boehringer Ingelheim, Bristol-Myers Squibb, Falk, Genfit, Gilead, Intercept, Janssen, MSD, Novartis, Phenex, Pliant, Regulus, and Shire, and received travel support from AbbVie, Falk, Gilead, and Intercept, as well as grants/research support from Albireo, Alnylam, Cymabay, Falk, Gilead, Intercept, MSD, Takeda, and UltraGenyx. He is also co-inventor of patents on the medical use of 24-norursodeoxycholic acid. MM served as a speaker and/or consultant and/or advisory board member for AbbVie, Collective Acumen, Gilead, Takeda, and W. L. Gore & Associates and received travel support from AbbVie and Gilead. TR served as a speaker and/or consultant and/or advisory board member for AbbVie, Bayer, Boehringer Ingelheim, Gilead, Intercept, MSD, Siemens, and W. L. Gore & Associates and received grants/research support from AbbVie, Boehringer Ingelheim, Gilead, Intercept, MSD, Myr Pharmaceuticals, Pliant, Philips, Siemens, and W. L. Gore & Associates as well as travel support from AbbVie, Boehringer Ingelheim, Gilead, and Roche.

Please refer to the accompanying ICMJE disclosure forms for further details.

Authors' contributions

Contributed to research design: LH, TR. Contributed to data acquisition: LH, BSim, MJ, DB, RP, BSch, LB, GS, MM, TR. Contributed to data analysis: LH, PW, MM, TR. Contributed to data interpretation: all authors. Drafted the manuscript: LH, TR. Critically revised the manuscript: all other authors.

Data availability statement

The data are available upon reasonable request to the corresponding author.

Supplementary data

Supplementary data to this article can be found online at https://doi.org/10.1016/j.jhepr.2023.100954.

References

Author names in bold designate shared co-first authorship

[1] Sepanlou SG, Safiri S, Bisignano C, et al. The global, regional, and national burden of cirrhosis by cause in 195 countries and territories, 1990–2017: a systematic analysis for the Global Burden of Disease Study 2017. Lancet Gastroenterol Hepatol 2020;5:245–266.

[2] D'Amico G, Garcia-Tsao G, Pagliaro L. Natural history and prognostic indicators of survival in cirrhosis: a systematic review of 118 studies. J Hepatol 2006;44:217–231.

[3] de Franchis R, Bosch J, Garcia-Tsao G, et al. Baveno VII – renewing consensus in portal hypertension. J Hepatol 2022;76:959–974.

[4] Mandorfer M, Aigner E, Cejna M, et al. Austrian consensus on the diagnosis and management of portal hypertension in advanced chronic liver disease (Billroth IV). Wien Klin Wochenschr 2023;135:493–523.

[5] **Costa D**, **Simbrunner B**, **Jachs M**, et al. Systemic inflammation increases across distinct stages of advanced chronic liver disease and correlates with decompensation and mortality. J Hepatol 2021;74:819–828.

[6] D'Amico G, Morabito A, D'Amico M, et al. Clinical states of cirrhosis and competing risks. J Hepatol 2018;68:563–576.

[7] Hartl L, Jachs M, Desbalmes C, et al. The differential activation of cardiovascular hormones across distinct stages of portal hypertension predicts clinical outcomes. Hepatol Int 2021;15:1160–1173.

[8] Jalan R, Saliba F, Pavesi M, et al. Development and validation of a prognostic score to predict mortality in patients with acute-on-chronic liver failure. J Hepatol 2014;61:1038–1047.

[9] Balcar L, Semmler G, Pomej K, et al. Patterns of acute decompensation in hospitalized patients with cirrhosis and course of acute-on-chronic liver failure. United Eur Gastroenterol J 2021;9:427–437.

[10] Moreau R, Jalan R, Gines P, et al. Acute-on-chronic liver failure is a distinct syndrome that develops in patients with acute decompensation of cirrhosis. Gastroenterology 2013;144:1426–1437. 1437.e1421–9.

[11] Trebicka J, Fernandez J, Papp M, et al. The PREDICT study uncovers three clinical courses of acutely decompensated cirrhosis that have distinct pathophysiology. J Hepatol 2020;73:842–854.

[12] Piantanida E, Ippolito S, Gallo D, et al. The interplay between thyroid and liver: implications for clinical practice. J Endocrinol Invest 2020;43:885–899.

[13] Targher G, Montagnana M, Salvagno G, et al. Association between serum TSH, free T4 and serum liver enzyme activities in a large cohort of unselected outpatients. Clin Endocrinol (Oxf) 2008;68:481–484.

[14] Mantovani A, Nascimbeni F, Lonardo A, et al. Association between primary hypothyroidism and nonalcoholic fatty liver disease: a systematic review and meta-analysis. Thyroid 2018;28:1270–1284.

[15] Guo Z, Li M, Han B, et al. Association of non-alcoholic fatty liver disease with thyroid function: a systematic review and meta-analysis. Dig Liver Dis 2018;50:1153–1162.

[16] Ritter MJ, Amano I, Hollenberg AN. Thyroid hormone signaling and the liver. Hepatology 2020;72:742–752.

[17] Borzio M, Caldara R, Borzio F, et al. Thyroid function tests in chronic liver disease: evidence for multiple abnormalities despite clinical euthyroidism. Gut 1983;24:631–636.

[18] Bernardi M, De Palma R, Trevisani F, et al. "Low T3 syndrome" in cirrhosis: effect of beta-blockade. Am J Gastroenterol 1989;84:727–731.

[19] Huang X, Jiang S, Fan X, et al. Low-free triiodothyronine is associated with poor prognosis of portal hypertension in cirrhosis. Eur J Gastroenterol Hepatol 2020;32:1358–1363.

[20] Reiberger T, Schwabl P, Trauner M, et al. Measurement of the hepatic venous pressure gradient and transjugular liver biopsy. J Vis Exp 2020;160:e58819.

[21] Scheiner B, Balcar L, Paternostro R, et al. Von Willebrand factor for outcome prediction within different clinical stages of advanced chronic liver disease. J Hepatol 2021;75:270–271.

[22] Zaccherini G, Weiss E, Moreau R. Acute-on-chronic liver failure: definitions, pathophysiology and principles of treatment. JHEP Rep 2021;3:100176.

[23] Ferlitsch A, Bota S, Paternostro R, et al. Evaluation of a new balloon occlusion catheter specifically designed for measurement of hepatic venous pressure gradient. Liver Int 2015;35:2115–2120.

[24] Schwabl P, Bota S, Salzl P, et al. New reliability criteria for transient elastography increase the number of accurate measurements for screening of cirrhosis and portal hypertension. Liver Int 2015;35:381–390.

[25] Fine JP, Gray RJ. A proportional hazards model for the subdistribution of a competing risk. J Am Stat Assoc 1999;94:496–509.

[26] Jachs M, Hartl L, Schaufler D, et al. Amelioration of systemic inflammation in advanced chronic liver disease upon beta-blocker therapy translates into improved clinical outcomes. Gut 2021;70:1758–1767.

[27] Punekar P, Sharma AK, Jain A. A study of thyroid dysfunction in cirrhosis of liver and correlation with severity of liver disease. Indian J Endocrinol Metab 2018;22:645–650.

[28] Vincken S, Reynaert H, Schiettecatte J, et al. Liver cirrhosis and thyroid function: friend or foe? Acta Clin Belg 2017;72:85–90.

[29] Fliers E, Boelen A. An update on non-thyroidal illness syndrome. J Endocrinol Invest 2021;44:1597–1607.

[30] Lee S, Farwell AP. Euthyroid sick syndrome. Compr Physiol 2016;6:1071–1080.

[31] Simbrunner B, Mandorfer M, Trauner M, et al. Gut–liver axis signaling in portal hypertension. World J Gastroenterol 2019;25:5897–5917.

[32] Tranah TH, Edwards LA, Schnabl B, et al. Targeting the gut–liver–immune axis to treat cirrhosis. Gut 2021;70:982–994.

[33] **Simbrunner B**, **Caparrós E**, **Neuwirth T**, et al. Bacterial translocation occurs early in cirrhosis and triggers a selective inflammatory response. Hepatol Int 2023;17:1045–1056.

[34] Boelen A, van der Spek AH, Bloise F, et al. Tissue thyroid hormone metabolism is differentially regulated during illness in mice. J Endocrinol 2017;233:25–36.

[35] Hartl L, Simbrunner B, Jachs M, et al. An impaired pituitary-adrenal signaling axis in stable cirrhosis is linked to worse prognosis. JHEP Rep 2023;5:100789.

[36] **Hartl L**, **Rumpf B**, **Domenig O**, et al. The systemic and hepatic alternative renin-angiotensin system is activated in liver cirrhosis, linked to endothelial dysfunction and inflammation. Sci Rep 2023;13:953.

[37] Mandorfer M, Schwabl P, Paternostro R, et al. Von Willebrand factor indicates bacterial translocation, inflammation, and procoagulant imbalance and predicts complications independently of portal hypertension severity. Aliment Pharmacol Ther 2018;47:980–988.

[38] Hegedüs L, Kastrup J, Feldt-Rasmussen U, et al. Serum thyroglobulin in acute and chronic liver disease. Clin Endocrinol (Oxf) 1983;19:231–237.

Journal of Hepatology, Volume *6*

Supplemental information

Lower free triiodothyronine (fT3) levels in cirrhosis are linked to systemic inflammation, higher risk of acute-on-chronic liver failure, and mortality

Lukas Hartl, Benedikt Simbrunner, Mathias Jachs, Peter Wolf, David Josef Maria Bauer, Bernhard Scheiner, Lorenz Balcar, Georg Semmler, Michael Schwarz, Rodrig Marculescu, Varius Dannenberg, Michael Trauner, Mattias Mandorfer, and Thomas Reiberger

Lower free triiodothyronine (fT3) levels in cirrhosis are linked to systemic inflammation, higher risk of acute-on-chronic liver failure, and mortality

Lukas Hartl, Benedikt Simbrunner, Mathias Jachs, Peter Wolf, David Josef Maria Bauer, Bernhard Scheiner, Lorenz Balcar, Georg Semmler, Michael Schwarz, Rodrig Marculescu, Varius Dannenberg, Michael Trauner, Mattias Mandorfer, Thomas Reiberger

Table of contents

Table S1……………………………………………………………………………2
Table S2……………………………………………………………………………3
Table S3……………………………………………………………………………4
Table S4……………………………………………………………………………5
Table S5……………………………………………………………………………6
Table S6……………………………………………………………………………7
Table S7……………………………………………………………………………9
Table S8………………………………………………………………………..…11
Table S9………………………………………………………………………..…13
Table S10…………………………………………………………………..…..14
Table S11…………………………………………………………………..…..15

Table-S1. Levels of pituitary-thyroid axis parameters, parameters of liver function and systemic inflammation in patients with different stages of advanced chronic liver disease

Parameter, Unit	EASL stage							p-value
	pACLD (n=10)	0 (n=33)	1 (n=42)	2 (n=44)	3 (n=12)	4 (n=97)	5 (n=59)	
**TSH, μIU x mL[1] (IQR)**	1.2 (0.7-2.3)	1.2 (1.0-1.7)	1.5 (0.8-2.1)	1.4 (1.1-2.1)	1.6 (0.8-2.7)	1.7 (1.2-2.4)	1.5 (1.0-2.3)	0.152
**Elevated TSH, n (%)**	0 (0.0%)	1 (3.0%)	2 (4.8%)	0 (0.0%)	0 (0.0%)	3 (3.1%)	3 (3.1%)	0.765
**fT4, ng x dL^{-1} (IQR)**	1.2 (1.0-1.3)	1.1 (1.1-1.3)	1.1 (1.0-1.2)	1.1 (1.0-1.3)	1.2 (1.0-1.3)	1.2 (1.0-1.3)	1.2 (1.1-1.3)	0.338
**Low fT4, n (%)**	0 (0.0%)	1 (3.0%)	0 (0.0%)	0 (0.0%)	0 (0.0%)	1 (1.0%)	1 (1.7%)	0.842
**fT3, pg x mL^{-1} (IQR)**	3.2 (3.1-3.5)	3.0 (2.7-3.3)	2.9 (2.5-3.2)	2.9 (2.6-3.2)	2.9 (2.3-3.3)	2.7 (2.3-3.0)	2.5 (2.1-2.8)	**<0.001**
**Low fT3, n (%)**	0 (0.0%)	2 (6.1%)	4 (9.5%)	3 (6.8%)	1 (8.3%)	12 (12.4%)	16 (27.1%)	**0.015**
**CTP score, points (IQR)**	5 (5-6)	5 (5-5)	5 (5-6)	5 (5-6)	5 (5-6)	7 (6-9)	8 (7-10)	**<0.001**
**MELD, points (IQR)**	8 (7-12)	8 (7-11)	9 (8-14)	11 (9-14)	11 (10-15)	14 (10-17)	14 (10-18)	**<0.001**
**Bilirubin, mg x dL^{-1} (IQR)**	0.7 (0.5-1.4)	0.8 (0.5-1.0)	0.8 (0.6-1.4)	1.3 (0.8-2.3)	1.0 (0.8-1.9)	1.3 (0.8-2.7)	1.7 (0.9-2.5)	**<0.001**
**Albumin, g x dL^{-1} (IQR)**	39.9 (37.0-41.5)	41.0 (38.9-43.0)	38.4 (34.3-42.4)	37.8 (35.6-40.4)	39.5 (37.7-40.7)	35.4 (31.1-38.5)	34.1 (29.9-37.7)	**<0.001**
**IL-6, ng x dL^{-1} (IQR) [1]**	4.6 (3.5-6.2)	4.2 (2.7-7.1)	5.8 (2.6-13.1)	6.1 (4.1-9.6)	6.6 (5.0-13.4)	10.5 (6.7-18.5)	13.5 (8.7-24.4)	**<0.001**
**CRP, mg x dL^{-1} (IQR)**	0.2 (0.0-0.2)	0.1 (0.1-0.3)	0.2 (0.1-0.7)	0.2 (0.1-0.3)	0.1 (0.1-0.3)	0.4 (0.2-0.8)	0.4 (0.2-0.7)	**<0.001**

[1] available in 286 patients (stage pACLD: n=10; 0: n=33; stage 1: n=41; stage 2: n=40; stage 3: n=12; stage 4: n=94; stage 5: n=56)

Table-S2. Levels of thyroid stimulating hormone (TSH) and free triiodothyronine (fT3) in different strata of body mass index (BMI) among patients with compensated (cACLD) and decompensated advanced chronic liver disease (dACLD)

	cACLD (n=129)				dACLD (n=168)			
**BMI strata**	**<25 kg/m^2**	**25-30 kg/m^2**	**>30 kg/m^2**	**p-value**	**<25 kg/m^2**	**25-30 kg/m^2**	**>30 kg/m^2**	**p-value**
**Number of patients, n (%)**	48 (37.2%)	39 (30.2%)	42 (32.6%)		81 (48.2%)	58 (34.5%)	29 (17.3%)	
**TSH, μIU/mL (IQR)**	1.3 (0.9-2.3)	1.3 (1.0-1.7)	1.4 (1.1-1.8)	0.844	1.7 (1.1-2.6)	1.4 (1.1-2.0)	2.0 (1.2-3.0)	0.514
**fT3, pg/mL (IQR)**	3.0 (2.6-3.3)	2.9 (2.6-3.1)	3.0 (2.7-3.3)	0.222	2.7 (2.2-3.0)	2.6 (2.4-3.0)	2.6 (2.2-2.9)	0.381

Table-S3. Correlations of pituitary-thyroid axis parameters, parameters of liver function, endothelial dysfunction, hyperdynamic circulation and systemic inflammation in patients advanced chronic liver disease

	fT3, pg x mL^{-1}		fT4, ng x dL^{-1}		TSH, µIU x mL^{-1}	
	rho	p-value	rho	p-value	rho	p-value
**TSH, µIU x mL^{-1}**	-0.152	0.009	-0.109	0.061	-	-
**fT4, ng x dL^{-1}**	0.000	0.998	-	-	-0.109	0.061
**fT3, pg x mL^{-1}**	-	-	0.000	0.998	-0.152	0.009
**MELD, points**	-0.448	<0.001	0.060	0.300	0.132	0.023
**Albumin, mg x L^{-1}**	0.434	<0.001	-0.012	0.835	-0.179	0.002
**HVPG, mmHg**	-0.300	<0.001	0.069	0.233	0.152	0.009
**VWF, %**	-0.357	<0.001	0.046	0.431	0.024	0.687
**proBNP, pg x mL^{-1}**	-0.385	<0.001	0.226	<0.001	0.107	0.070
**IL-6, ng x dL^{-1} (IQR)** [3]	-0.496	<0.001	0.146	0.013	0.222	<0.001
**CRP, mg x dL^{-1}**	-0.356	<0.001	0.188	0.001	0.113	0.052

Table-S4. Impact of parameters of hepatic dysfunction, kidney function and systemic inflammation on (i) free triiodothyronine (fT3) and (ii) thyroid stimulating hormone (TSH) levels. Univariate and multivariate multivariate linear regression models are shown.

Parameter, Unit	Univariate (unadjusted) analysis			Multivariate (adjusted) analysis		
**(i) fT3, pg/mL**	**B**	**95%CI**	**p-value**	**aB**	**95%CI**	**p-value**
**Age, 10 years**	-0.10	-0.16 - -0.03	**0.005**	-0.11	-0.17 – -0.05	**<0.001**
**Sex (male)**	0.10	-0.08 - 0.28	0.276	-	-	-
**Body Mass Index, kg x m^{-2}**	0.01	-0.01 - 0.02	0.621	-0.01	-0.02 - 0.01	0.495
**Child Turcotte Pugh score, points**	-0.16	-0.20 - -0.12	**<0.001**	-0.15	-0.19 - -0.11	**<0.001**
**Creatinine, mg x dL^{-1}**	-0.08	-0.17 - 0.01	0.074	-0.06	-0.15 – 0.02	0.129
**Sodium, mmol x L^{-1}**	0.04	0.02 - 0.06	**<0.001**	-0.01	-0.04 - 0.01	0.279
**HVPG, mmHg**	-0.03	-0.04 - -0.02	**<0.001**	-0.01	-0.02 - 0.00	0.161
**C-reactive protein, mg x dL^{-1}**	-0.42	-0.59 - -0.25	**<0.001**	-0.17	-0.34 - -0.01	**0.048**
**(ii) TSH, µIU/mL**	**B**	**95%CI**	**p-value**	**aB**	**95%CI**	**p-value**
**Age, 10 years**	-0.01	-0.17 - 0.17	0.955	-	-	-
**Sex (male)**	-0.21	-0.66 - 0.25	0.371	-	-	-
**Body Mass Index, kg x m^{-2}**	-0.01	-0.05 – 0.03	0.690	0.00	-0.04 – 0.04	0.935
**Child Turcotte Pugh score, points**	0.07	-0.04 - 0.18	0.190	0.02	-0.11 - 0.14	0.779
**Creatinine, mg x dL^{-1}**	0.07	-0.16 - 0.30	0.533	-	-	-
**Sodium, mmol x L^{-1}**	0.02	-0.03 - 0.08	0.426	-	-	-
**HVPG, mmHg**	0.03	-0.01 - 0.06	0.113	0.02	-0.02 - 0.05	0.318
**C-reactive protein, mg x dL^{-1}**	0.45	0.01 - 0.89	**0.046**	0.45	0.01 - 0.89	**0.046**

Table-S5. Impact of parameters of hepatic dysfunction, kidney function and systemic inflammation on (i) free triiodothyronine (fT3) and (ii) thyroid stimulating hormone (TSH) levels in patients with decompensated advanced chronic liver disease (dACLD).

Univariate and multivariate multivariate linear regression models are shown.

Parameter, Unit	Univariate (unadjusted) analysis			Multivariate (adjusted) analysis		
**(i) fT3, pg/mL**	**B**	**95%CI**	**p-value**	**aB**	**95%CI**	**p-value**
**Age, 10 years**	-0.04	-0.11 - 0.02	0.200	-	-	-
**Sex (male)**	0.13	-0.05 - 0.31	0.164	-	-	-
**Body Mass Index, kg x m^{-2}**	0.00	-0.02 - 0.02	0.950	-0.01	-0.02 - 0.01	0.465
**Child Turcotte Pugh score, points**	-0.13	-0.17 - -0.09	**<0.001**	-0.11	-0.15 - -0.07	**<0.001**
**Creatinine, mg x dL^{-1}**	-0.10	-0.18 - -0.01	**0.023**	-0.08	-0.15 – 0.00	**0.049**
**Sodium, mmol x L^{-1}**	0.02	0.00 - 0.04	0.058	-0.01	-0.03 - 0.01	0.178
**HVPG, mmHg**	-0.02	-0.04 - -0.01	**0.008**	-0.01	-0.02 - 0.00	0.167
**C-reactive protein, mg x dL^{-1}**	-0.32	-0.47 - -0.18	**<0.001**	-0.19	-0.33 - -0.05	**0.008**
**(ii) TSH, µIU/mL**	**B**	**95%CI**	**p-value**	**aB**	**95%CI**	**p-value**
**Age, 10 years**	0.00	-0.28 - 0.27	0.978	-	-	-
**Sex (male)**	-0.46	-1.21 - 0.28	0.223	-	-	-
**Body Mass Index, kg x m^{-2}**	-0.01	-0.08 – 0.06	0.839	0.00	-0.07 – 0.07	0.912
**Child Turcotte Pugh score, points**	0.03	-0.14 - 0.21	0.719	0.02	-0.16 - 0.20	0.799
**Creatinine, mg x dL^{-1}**	0.10	-0.25 - 0.45	0.571	-	-	-
**Sodium, mmol x L^{-1}**	0.07	-0.02 - 0.14	0.129	-	-	-
**HVPG, mmHg**	0.02	-0.05 - 0.08	0.562	0.02	-0.05 - 0.08	0.562
**C-reactive protein, mg x dL^{-1}**	0.43	-0.21 - 1.06	0.185	-	-	-

Table-S6. Impact of thyroid stimulating hormone (TSH) on the risk of (i) decompensation/further decompensation, (ii) acute-on-chronic liver failure (ACLF) and (iii) liver-related death. Univariate and multivariate multivariate competing risk regression models are shown. Etiological cure, hepatocellular carcinoma, liver transplantation and non-liver-related death were considered as competing risks.

**Parameter, Unit**	**Univariate (unadjusted) analysis**			**Multivariate (adjusted) analysis**		
**(i) Outcome: (further) decompensation**	**sHR**	**95%CI**	**p-value**	**asHR**	**95%CI**	**p-value**
**TSH, µIU x mL[1]**	1.02	0.94-1.10	0.660	1.00	0.90-1.10	0.930
**Age, 10 years**	0.98	0.81-1.19	0.840	-	-	-
**Sex (male)**	0.90	0.54-1.51	0.690	-	-	-
**Body Mass Index, kg x m^{-2}**	0.99	0.93-1.05	0.720	1.03	0.96-1.10	0.470
**Child Turcotte Pugh score, points**	1.26	1.13-1.39	**<0.001**	1.19	1.03-1.37	**0.019**
**Creatinine, mg x dL^{-1}**	0.83	0.60-1.16	0.280	-	-	-
**Sodium, mmol x L^{-1}**	0.93	0.89-0.98	**0.009**	1.01	0.94-1.08	0.810
**HVPG, mmHg**	1.10	1.07-1.14	**<0.001**	1.09	1.04-1.14	**<0.001**
**C-reactive protein, mg x dL^{-1}**	1.49	1.01-2.19	**0.042**	0.85	0.52-1.39	0.530
**(ii) Outcome: ACLF**	**sHR**	**95%CI**	**p-value**	**asHR**	**95%CI**	**p-value**
**TSH, µIU x mL[1]**	1.05	0.94-1.17	0.400	1.07	0.94-1.22	0.290
**Age, 10 years**	1.27	0.83-1.94	0.270	-	-	-
**Sex (male)**	1.02	0.39-2.65	0.970	-	-	-
**Body Mass Index, kg x m^{-2}**	0.95	0.78-1.15	0.610	1.01	0.81-1.26	0.940
**Child Turcotte Pugh score, points**	1.54	1.28-1.86	**<0.001**	1.36	1.07-1.74	**0.013**
**Creatinine, mg x dL^{-1}**	1.23	1.06-1.43	**0.007**	1.00	0.84-1.20	0.970
**Sodium, mmol x L^{-1}**	0.85	0.79-0.92	**<0.001**	0.93	0.85-1.03	0.170
**HVPG, mmHg**	1.07	0.92-1.25	0.390	1.09	1.00-1.20	0.063
**C-reactive protein, mg x dL^{-1}**	2.21	1.21-4.02	**0.009**	0.99	0.51-1.94	0.980

(iii) Outcome: Liver-related death	sHR	95%CI	p-value	asHR	95%CI	p-value
**TSH, µIU x mL[1]**	1.06	0.96-1.16	0.250	1.08	0.95-1.24	0.250
**Age, 10 years**	1.22	0.75-2.01	0.420	-	-	-
**Sex (male)**	0.78	0.27-2.30	0.650	-	-	-
**Body Mass Index, kg x m^{-2}**	0.91	0.79-1.05	0.210	0.98	0.81-1.18	0.810
**Child Turcotte Pugh score, points**	1.63	1.26-2.09	**<0.001**	1.42	0.99-2.04	0.054
**Creatinine, mg x dL^{-1}**	1.03	0.86-1.22	0.780	-	-	-
**Sodium, mmol x L^{-1}**	0.83	0.75-0.91	**<0.001**	0.90	0.79-1.04	0.160
**HVPG, mmHg**	1.15	1.07-1.24	**<0.001**	1.11	0.99-1.23	0.073
**C-reactive protein, mg x dL^{-1}**	2.21	1.12-4.35	**0.022**	0.84	0.38-1.84	0.660

Table-S7. Impact of free thyroxin (fT4) on the risk of (i) decompensation/further decompensation, (ii) acute-on-chronic liver failure (ACLF) and (iii) liver-related death. Univariate and multivariate multivariate competing risk regression models are shown. Etiological cure, hepatocellular carcinoma, liver transplantation and non-liver-related death were considered as competing risks.

**Parameter of interest**	**Univariate (unadjusted) analysis**			**Multivariate (adjusted) analysis**		
**(i) Outcome: (further) decompensation**	**sHR**	**95%CI**	**p-value**	**asHR**	**95%CI**	**p-value**
**fT4, ng x dL^{-1}**	1.12	0.52-2.42	0.780	0.92	0.38-2.23	0.860
**Age, 10 years**	0.98	0.81-1.19	0.840	-	-	-
**Sex (male)**	0.90	0.54-1.51	0.690	-	-	-
**Body Mass Index, kg x m^{-2}**	0.99	0.93-1.05	0.720	1.03	0.95-1.10	0.480
**Child Turcotte Pugh score, points**	1.26	1.13-1.39	**<0.001**	1.18	1.02-1.37	**0.024**
**Creatinine, mg x dL^{-1}**	0.83	0.60-1.16	0.280	-	-	-
**Sodium, mmol x L^{-1}**	0.93	0.89-0.98	**0.009**	1.01	0.94-1.08	0.850
**HVPG, mmHg**	1.10	1.07-1.14	**<0.001**	1.09	1.04-1.14	**<0.001**
**C-reactive protein, mg x dL^{-1}**	1.49	1.01-2.19	**0.042**	0.86	0.51-1.46	0.580
**(ii) Outcome: ACLF**	**sHR**	**95%CI**	**p-value**	**asHR**	**95%CI**	**p-value**
**fT4, ng x dL^{-1}**	1.33	0.31-5.64	0.700	0.42	0.08-2.16	0.300
**Age, 10 years**	1.27	0.83-1.94	0.270	-	-	-
**Sex (male)**	1.02	0.39-2.65	0.970	-	-	-
**Body Mass Index, kg x m^{-2}**	0.95	0.78-1.15	0.610	1.00	0.79-1.26	0.990
**Child Turcotte Pugh score, points**	1.54	1.28-1.86	**<0.001**	1.34	1.03-1.73	**0.027**
**Creatinine, mg x dL^{-1}**	1.23	1.06-1.43	**0.007**	0.97	0.80-1.17	0.710
**Sodium, mmol x L^{-1}**	0.85	0.79-0.92	**<0.001**	0.92	0.83-1.02	0.120
**HVPG, mmHg**	1.07	0.92-1.25	0.390	1.10	1.00-1.20	0.051

**C-reactive protein, mg x dL^{-1}**	2.21	1.21-4.02	**0.009**	1.17	0.56-2.45	0.680
**(iii) Outcome: Liver-related death**	**sHR**	**95%CI**	**p-value**	**asHR**	**95%CI**	**p-value**
**fT4, ng x dL^{-1}**	1.61	0.32-8.21	0.560	0.560	0.06-5.14	0.610
**Age, 10 years**	1.22	0.75-2.01	0.420	-	-	-
**Sex (male)**	0.78	0.27-2.30	0.650	-	-	-
**Body Mass Index, kg x m^{-2}**	0.91	0.79-1.05	0.210	0.97	0.82-1.15	0.710
**Child Turcotte Pugh score, points**	1.63	1.26-2.09	**<0.001**	1.40	0.93-2.10	0.110
**Creatinine, mg x dL^{-1}**	1.03	0.86-1.22	0.780	-	-	-
**Sodium, mmol x L^{-1}**	0.83	0.75-0.91	**<0.001**	0.90	0.76-1.06	0.200
**HVPG, mmHg**	1.15	1.07-1.24	**<0.001**	1.11	0.99-1.24	0.070
**C-reactive protein, mg x dL^{-1}**	2.21	1.12-4.35	**0.022**	0.96	0.35-2.64	0.940

Table-S8. Patient characteristics according to low vs. non-low free triiodothyronine (fT3) levels

Patient characteristics	Patients with low fT3 (n=38)	Patients without low fT3 (n=259)	p-value
**Sex, male/female (% male)**	18/20 (47.4%)	181/78 (69.9%)	**0.006**
**Age, years (IQR)**	55.3 (47.1-63.9)	56.0 (48.1-63.8)	0.915
**BMI, kg x m^{-2} (IQR)**	23.3 (20.7-26.3)	26.4 (23.5-30.2)	**0.003**
**Etiology**			0.156
**ALD, n (%)**	24 (63.2%)	136 (52.5%)	
**Viral hepatitis, n (%)**	2 (5.3%)	47 (18.1%)	
**MASH, n (%)**	2 (5.3%)	33 (12.7%)	
**Cholestatic, n (%)**	3 (7.9%)	18 (7.0)	
**AIH, n (%)**	2 (5.3%)	7 (2.7%)	
**Other, n (%)**	5 (13.2%)	18 (7.0%)	
**Decompensated ACLD, n (%)**	29 (76.3%)	139 (53.7%)	**0.009**
**Ascites, n (%)**	28 (73.7%)	116 (44.8%)	**<0.001**
**Refractory ascites, n (%)**	11 (28.9%)	16 (6.2%)	**<0.001**
**Hepatic encephalopathy, n (%)**	13 (34.2%)	41 (15.8%)	**0.006**
**Variceal bleeding, n (%)**	5 (13.2%)	30 (11.6%)	0.779
**EASL stage**			**0.015**
**probable ACLD, n (%)**	0 (0.0%)	10 (3.9%)	
**0, n (%)**	2 (5.3%)	31 (12.0%)	
**1, n (%)**	4 (10.5%)	38 (14.7%)	
**2, n (%)**	3 (7.9%)	41 (15.8%)	
**3, n (%)**	1 (2.6%)	11 (4.2%)	
**4, n (%)**	12 (31.6%)	85 (32.8%)	
**5, n (%)**	16 (42.1%)	43 (16.6%)	
**MELD, points (IQR)**	17 (14-19)	11 (9-14)	**<0.001**
**CTP score, points (IQR)**	9 (8-10)	6.0 (5-8)	**<0.001**

**CTP stage**			**<0.001**
**A, n (%)**	4 (10.6%)	155 (59.8%)	
**B, n (%)**	20 (52.6%)	82 (31.7%)	
**C, n (%)**	14 (36.8%)	22 (8.5%)	
**Bilirubin, mg x dL^{-1} (IQR)**	2.6 (1.7-4.3)	1.0 (0.7-2.0)	**<0.001**
**Albumin, g x dL^{-1} (IQR)**	31.9 (27.4-35.9)	37.4 (33.6-40.4)	**<0.001**
**INR, units (IQR)**	1.6 (1.3-1.8)	1.4 (1.2-1.5)	**0.039**
**Sodium, mmol x L^{-1} (IQR)**	136.0 (134.0-138.0)	139.0 (137.0-141.0)	**0.001**
**HVPG, mmHg (IQR)**	19 (15-24)	16 (11-20)	0.315
**MAP, mmHg (IQR)**	91 (82-106)	101 (91-111)	0.059
**LSM, kPa (IQR)**	57.3 (31.4-75.0)	35.6 (21.6-59.5)	**0.023**
**TSH, µIU x mL-1 (IQR)**	2.0 (1.3-2.6)	1.4 (1.0-2.2)	**0.023**
**fT3, pg x mL-1 (IQR)**	1.9 (1.7-2.0)	2.9 (2.6-3.2)	**<0.001**
**fT4, ng x dL-1 (IQR)**	1.2 (1.1-1.3)	1.2 (1.0-1.3)	0.612
**IL-6, ng x dL^{-1} (IQR)**	22.0 (13.3-27.4)	7.3 (4.3-13.1)	**<0.001**
**WBC, G x L^{-1} (IQR)**	4.6 (3.3-6.5)	4.8 (3.5-6.3)	0.999
**CRP, mg x dL^{-1} (IQR)**	0.5 (0.2-1.5)	0.2 (0.1-0.6)	**<0.001**

Table-S9. Clinical outcomes of patients according to low vs. non-low free triiodothyronine (fT3)

Clinical outcomes	Patients with low fT3 (n=38)	Patients with non-low fT3 (n=259)	p-value
**Follow-up time, days (IQR)**	292.0 (154.0-604.0)	427.0 (156.0-823.0)	0.233
**Decompensation event, n (%)**	14 (36.8%)	61 (23.6%)	0.078
**Ascitic complication, n (%)**	4 (10.5%)	39 (15.1%)	0.458
**Variceal bleeding, n (%)**	3 (7.9%)	10 (3.9%)	0.256
**Hepatic encephalopathy, n (%)**	2 (5.3%)	37 (14.3%)	0.124
**ACLF, n (%)**	11 (28.9%)	15 (5.8%)	**<0.001**
**Bacterial infections, n (%)[1]**	5 (13.5%)	33 (13.2%)	0.958
**SBP, n (%)**	3 (7.9%)	5 (1.9%)	**0.034**
**Acute kidney injury, n (%)[1]**	5 (13.5%)	19 (7.6%)	0.225
**HCC, n (%)**	0 (0.0%)	10 (3.9%)	0.218
**TIPS implantation, n (%)**	4 (10.5%)	13 (5.0%)	0.172
**Liver transplantation, n (%)**	4 (10.5%)	15 (5.8%)	0.265
**Death, n (%)**	11 (28.9%)	22 (8.5%)	**<0.001**
**Liver-related death, n (%)**	11 (28.9%)	9 (3.5%)	**<0.001**

[1] information on bacterial infections and acute kidney injury available in n=287 patients

Table-S10. Cumulative incidences of clinical events in patients with ACLD and low free triiodothyronine (fT3) and non-low fT3 levels

Clinical outcomes	Parameter	0.5 years	1 year	1.5 years	2 years	p-value
**Decompensation/further decompensation**	Low fT3	31.7%	31.7%	31.7%	41.0%	**0.039**
	Non-low fT3	12.1%	16.0%	20.2%	23.9%	
**ACLF**	Low fT3	11.1%	11.1%	17.4%	30.6%	**<0.001**
	Non-low fT3	0.0%	2.8%	4.1%	4.9%	
**Liver-related death**	Low fT3	15.2%	18.6%	18.6%	28.4%	**<0.001**
	Non-low fT3	0.0%	1.8%	1.8%	2.7%	

Table-S11. Impact of low free triiodothyronine (fT3) on the risk of (i) decompensation/further decompensation, (ii) acute-on-chronic liver failure (ACLF) and (iii) liver-related death. Univariate and multivariate multivariate competing risk regression models are shown. Etiological cure, hepatocellular carcinoma, liver transplantation and non-liver-related death were considered as competing risks.

**Parameter, Unit**	**Univariate (unadjusted) analysis**			**Multivariate (adjusted) analysis**		
**(i) Outcome: (further) decompensation**	**sHR**	**95%CI**	**p-value**	**asHR**	**95%CI**	**p-value**
**Low fT3, yes**	1.93	0.99-3.78	0.054	1.33	0.58-3.05	0.510
**Age, 10 years**	0.98	0.81-1.19	0.840	-	-	-
**Sex (male)**	0.90	0.54-1.51	0.690	-	-	-
**Body Mass Index, kg x m^{-2}**	0.99	0.93-1.05	0.720	1.03	0.96-1.11	0.400
**Child Turcotte Pugh score, points**	1.26	1.13-1.39	**<0.001**	1.17	1.01-1.36	**0.041**
**Creatinine, mg x dL^{-1}**	0.83	0.60-1.16	0.280	-	-	-
**Sodium, mmol x L^{-1}**	0.93	0.89-0.98	**0.009**	1.01	0.94-1.08	0.810
**HVPG, mmHg**	1.10	1.07-1.14	**<0.001**	1.09	1.05-1.14	**<0.001**
**C-reactive protein, mg x dL^{-1}**	1.49	1.01-2.19	**0.042**	0.83	0.50-1.37	0.470
**(ii) Outcome: ACLF**	**sHR**	**95%CI**	**p-value**	**asHR**	**95%CI**	**p-value**
**Low fT3, yes**	7.89	3.29-18.90	**<0.001**	4.58	1.77-11.87	**0.002**
**Age, 10 years**	1.27	0.83-1.94	0.270	-	-	-
**Sex (male)**	1.02	0.39-2.65	0.970	-	-	-
**Body Mass Index, kg x m^{-2}**	0.95	0.78-1.15	0.610	1.03	0.95-1.12	0.450
**Child Turcotte Pugh score, points**	1.54	1.28-1.86	**<0.001**	1.26	0.98-1.61	0.071
**Creatinine, mg x dL^{-1}**	1.23	1.06-1.43	**0.007**	0.89	0.71-1.12	0.340
**Sodium, mmol x L^{-1}**	0.85	0.79-0.92	**<0.001**	0.91	0.82-1.01	0.085
**HVPG, mmHg**	1.07	0.92-1.25	0.390	1.09	1.01-1.17	**0.020**

**C-reactive protein, mg x dL^{-1}**	2.21	1.21-4.02	**0.009**	0.91	0.45-1.83	0.780
**(iii) Outcome: Liver-related death**	**sHR**	**95%CI**	**p-value**	**asHR**	**95%CI**	**p-value**
**Low fT3, yes**	12.80	4.55-35.80	**<0.001**	6.80	2.05-22.56	**0.002**
**Age, 10 years**	1.22	0.75-2.01	0.420	-	-	-
**Sex (male)**	0.78	0.27-2.30	0.650	-	-	-
**Body Mass Index, kg x m^{-2}**	0.91	0.79-1.05	0.210	1.04	0.89-1.21	0.640
**Child Turcotte Pugh score, points**	1.63	1.26-2.09	**<0.001**	1.22	0.82-1.80	0.330
**Creatinine, mg x dL^{-1}**	1.03	0.86-1.22	0.780	-	-	-
**Sodium, mmol x L^{-1}**	0.83	0.75-0.91	**<0.001**	0.89	0.76-1.04	0.140
**HVPG, mmHg**	1.15	1.07-1.24	**<0.001**	1.11	1.02-1.21	**0.017**
**C-reactive protein, mg x dL^{-1}**	2.21	1.12-4.35	**0.022**	0.67	0.31-2.12	0.670
